# Biomimetic Microstructured Scaffold with Release of Re‐Modified Teriparatide for Osteoporotic Tendon‐to‐Bone Regeneration via Balancing Bone Homeostasis

**DOI:** 10.1002/advs.202500144

**Published:** 2025-03-17

**Authors:** Chengzhong Xu, Sijie Qiu, Zhigen Yuan, Chongyin Qiu, Wenyu Xu, Jialiang Guo, Gen Wen, Shuai Liu, Wenjuan Yan, Haibing Xu, Honghao Hou, Dehong Yang

**Affiliations:** ^1^ Department of Orthopaedics‐Spine Surgery Nanfang Hospital Southern Medical University 1838 North Guangzhou Ave Guangzhou 510515 P. R. China; ^2^ Guangdong Provincial Key Laboratory of Construction and Detection in Tissue Engineering School of Basic Medical Sciences Southern Medical University Guangzhou 510515 P. R. China; ^3^ Department of Stomatology Nanfang Hospital Southern Medical University Guangzhou 510515 P. R. China; ^4^ Guangdong‐Hong Kong‐Macao Greater Bay Area Center for Brain Science and Brain‐Inspired Intelligence Department of Neurobiology School of Basic Medical Sciences Southern Medical University Guangzhou 510515 P. R. China

**Keywords:** biomimetic microstructure, bone homeostasis, osteoporosis, recombinant human PTH, tendon‐to‐bone interfaces

## Abstract

Osteoporotic tendon‐to‐bone interface healing is challenging, with a high surgical repair failure rate of up to 68%. Conventional tissue engineering approaches have primarily focused on promoting interface healing by stimulating regeneration in either the tendon or bone. However, these methods often fall short of achieving optimal therapeutic outcomes due to their neglect of balancing bone homeostasis and remodeling the microstructure at the osteoporotic tendon‐to‐bone interface. Herein, a series of site‐specific functional modifications are carried out on teriparatide to develop recombinant human parathyroid hormone (R‐PTH). A biomimetic microstructured reconstruction scaffold (BMRP) is constructed using a decalcified mussel shell scaffold, pre‐gel, and R‐PTH. The BMRP mimics the microstructures of the native tendon‐to‐bone interface and restores the original structure of the interface tissue by repairing injured cells, balancing bone homeostasis, and remodeling the microstructure of the osteoporotic tendon‐to‐bone interface. In an osteoporotic rotator cuff tear model, BMRP is in situ implanted at the injured site, resulting in structural reconstruction and functional recovery. The BMRP demonstrates excellent repair effects, representing a novel therapeutical alternative for treating osteoporotic tendon‐to‐bone injury potential for clinical application.

## Graphical abstract

This study develops RPTH by re‐modifying teriparatide and designs a biomimetic microstructured scaffold (BMRP) loaded with RPTH to address osteoporotic tendon‐to‐bone interface repair. The BMRP restores tissue architecture by balancing bone homeostasis and remodeling the microstructure, achieving functional recovery in an osteoporotic rotator cuff tear model, offering a promising clinical solution.

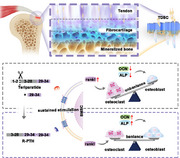

## 1 Introduction

Tendon‐to‐bone interface injuries are a pressing clinical challenge, with rotator cuff tears (RCT) being one type of such injury that can lead to severe shoulder disability.^[^ ^1^, ^2^ ^]^ Osteoporosis, as an independent risk factor, significantly influences the occurrence and progression of RCT. With the aging of the population, the number of patients with osteoporosis is increasing yearly, and compared to ordinary patients, the risk of re‐tear after rotator cuff repair (RCR) in osteoporosis patients increases by seven times.^[^ ^3^, ^4^ ^]^ Various treatment methods, such as 3D printed scaffolds, cell transplantation, and anti‐osteoporosis drugs, have been used for the repair of the tendon‐to‐bone interface, promoting healing to varying degrees.^[^ ^5^, ^6^, ^7^ ^]^ However, during the later stages of healing, the interface tissue is often replaced by fibrous scar tissue, making it difficult to restore its original function and the native microstructure of the interface.^[^ ^2^ ^]^ Therefore, effective methods for treating osteoporotic RCT and preventing secondary tears have yet to be fully met but are urgently needed.

Regulating the balance of bone homeostasis and biomimetic reconstructing of the microstructure in osteoporotic RCT play crucial roles in determining the fate of the affected interface tissue.^[^ ^8^ ^]^ Effective treatment and prevention of re‐tears depend on managing the complex interactions between bone marrow mesenchymal stem cells (BMSCs), tendon‐derived stem cells (TDSCs), and the tendon‐to‐bone interface bone homeostasis. osteoporotic RCT is primarily treated through rotator cuff repair surgery, but ongoing research aims to promote healing of the injury. Previous studies have typically focused on regulating biological factors such as osteogenic genes and key proteins within the tendon‐to‐bone interface for treating RCT.^[^ ^9^, ^10^, ^11^, ^12^, ^13^ ^]^ Given the unique characteristics of the osteoporotic tendon‐to‐bone interface, the equilibrium of bone homeostasis and the remodeling of microstructure are complementary and interdependent. Simply modulating osteogenesis or inhibiting osteoclast activity while neglecting bone homeostasis and microstructural remodeling at the interface may only lead to limited and transient therapeutic effects, failing to integrate the soft tendon tissue with the hard bone tissue effectively.

Under its unique physical and chemical conditions, an imbalance in bone metabolism and the disruption of the gradient‐mineralized bone microstructure contribute to an increased likelihood of RCT. The destruction of the osteoporotic tendon‐to‐bone interface microstructure, along with the infiltration of inflammatory cells, further leads to a disordered bone metabolic microenvironment at the interface.^[^ ^14^, ^15^, ^16^ ^]^ Excessive production of reactive oxygen species (ROS) results in injury to BMSCs and TDSCs at the interface, leading to decreased cell viability, loss of migratory capacity, or various forms of cell death. Currently, researchers are utilizing mono‐phasic or multi‐phasic scaffolds primarily to regulate osteogenesis or tenogenesis.^[^ ^17^, ^18^, ^19^, ^20^ ^]^ However, these scaffolds do not effectively mimic the complex microstructure of the interface and lack a comprehensive 3D approach that addresses cell migration, bone homeostasis, and biomimetic microstructure. Teriparatide (PTH1‐34) is a commonly used anti‐osteoporotic drug in clinical settings, however, high doses or continuous local stimulation can overactivate the osteoclasts and increase bone resorption.^[^ ^21^, ^22^ ^]^ Therefore, Teriparatide, as a “double‐edged sword,” faces the challenge of tricky delicate balancing bone formation and bone resorption. To avoid this drawback of Teriparatide, we have conducted a series of site‐specific functional re‐modifications of the polypeptide, leading to the development and design of recombinant human parathyroid hormone (R‐PTH).^[^ ^23^ ^]^ However, due to the short half‐life and easy deactivation of peptides in the body, achieving long‐lasting and stable effects of R‐PTH at the osteoporotic tendon‐to‐bone interface remains a challenge. Restoring the migratory capacity of injured cells, balancing bone homeostasis, and remodeling the bone microstructure are key bottlenecks in treating the osteoporotic tendon‐to‐bone interface.

**Figure 1:**
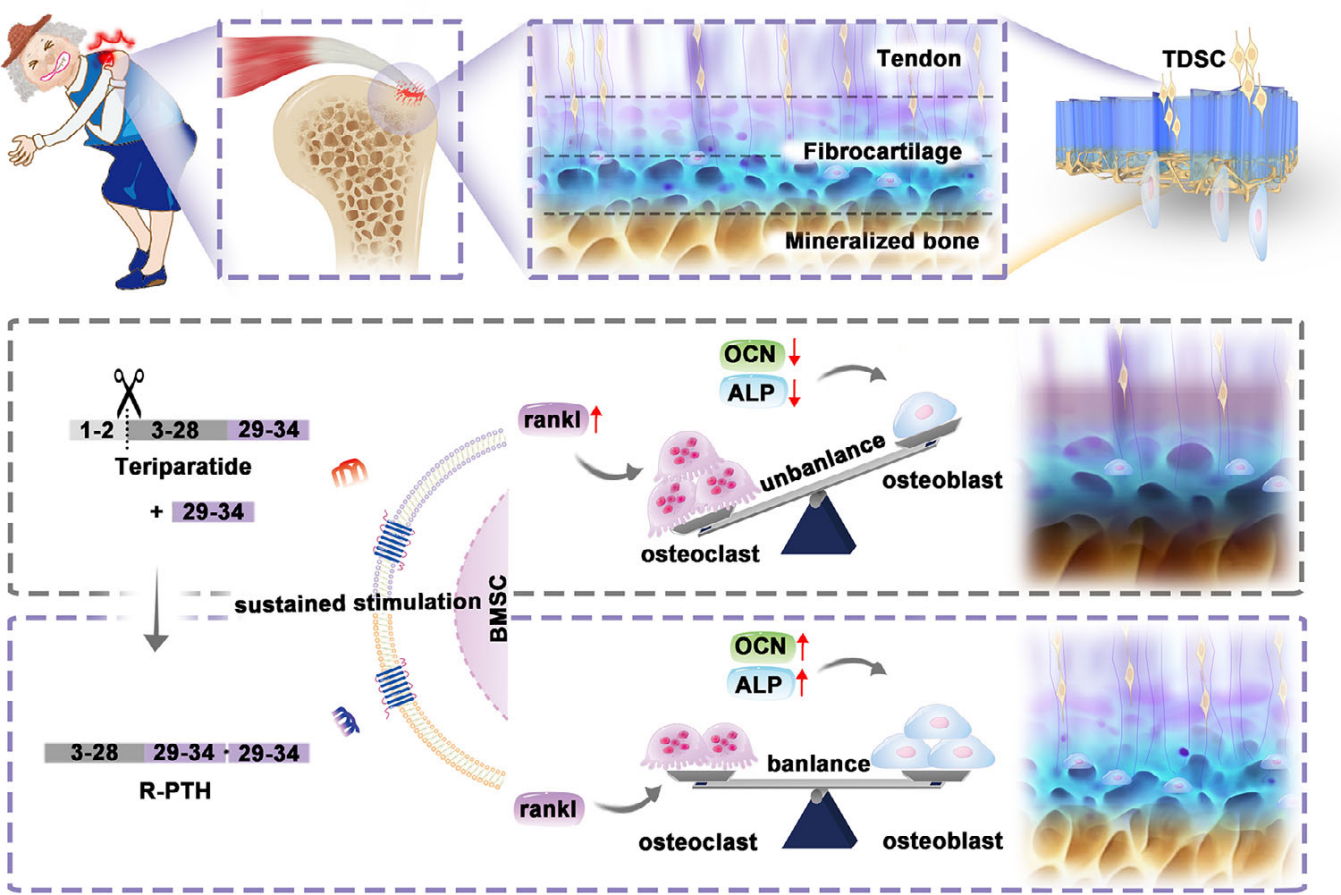
Construction of biomimetic microstructured scaffold and its promotion of osteoporotic tendon‐to‐bone interface regeneration by balancing bone homeostasis and remodeling microstructure.

In this work, a biomimetic microstructured scaffold loaded with R‐PTH (BMRP) was developed to promote the reconstruction and regeneration of the injured tendon‐to‐bone interface. Specifically, a glycosaminoglycan‐based 3D porous scaffold, featuring a microstructure similar to that of bone tissue, was obtained through the demineralization of natural mussel shells. On this basis, a pre‐gel solution was casted on the glycosaminoglycan scaffold to construct an oriented gel layer through the ice‐templating method, mimicking the aligned microstructure of tendon fibers. The BMRP scaffold effectively repairs injured cells and promotes the migration of tendon cells to the bone surface, facilitating the integration of tendon cells with bone cells. The BMRP scaffold releases R‐PTH in a controlled manner, regulating bone formation and resorption at the osteoporotic tendon‐to‐bone interface to remodel the bone microstructure. Moreover, the effects of continuous stimulation of BMSCs by Teriparatide or R‐PTH on the differentiation of osteoblasts and osteoclasts were further investigated, exploring the mechanism by which R‐PTH balances bone homeostasis at the osteoporotic tendon‐to‐bone interface (**Figure** **1**). The construction of biomimetic microstructured scaffolds to balance bone homeostasis at the osteoporotic tendon‐to‐bone interface provides a promising strategy for the integrated regeneration of soft and hard tissue interfaces.

## 2 Result and Discussion

### 2.1 Construction and Characterization of Biomimetic Tendon‐to‐Bone Microstructured Scaffold

**Figure 2:**
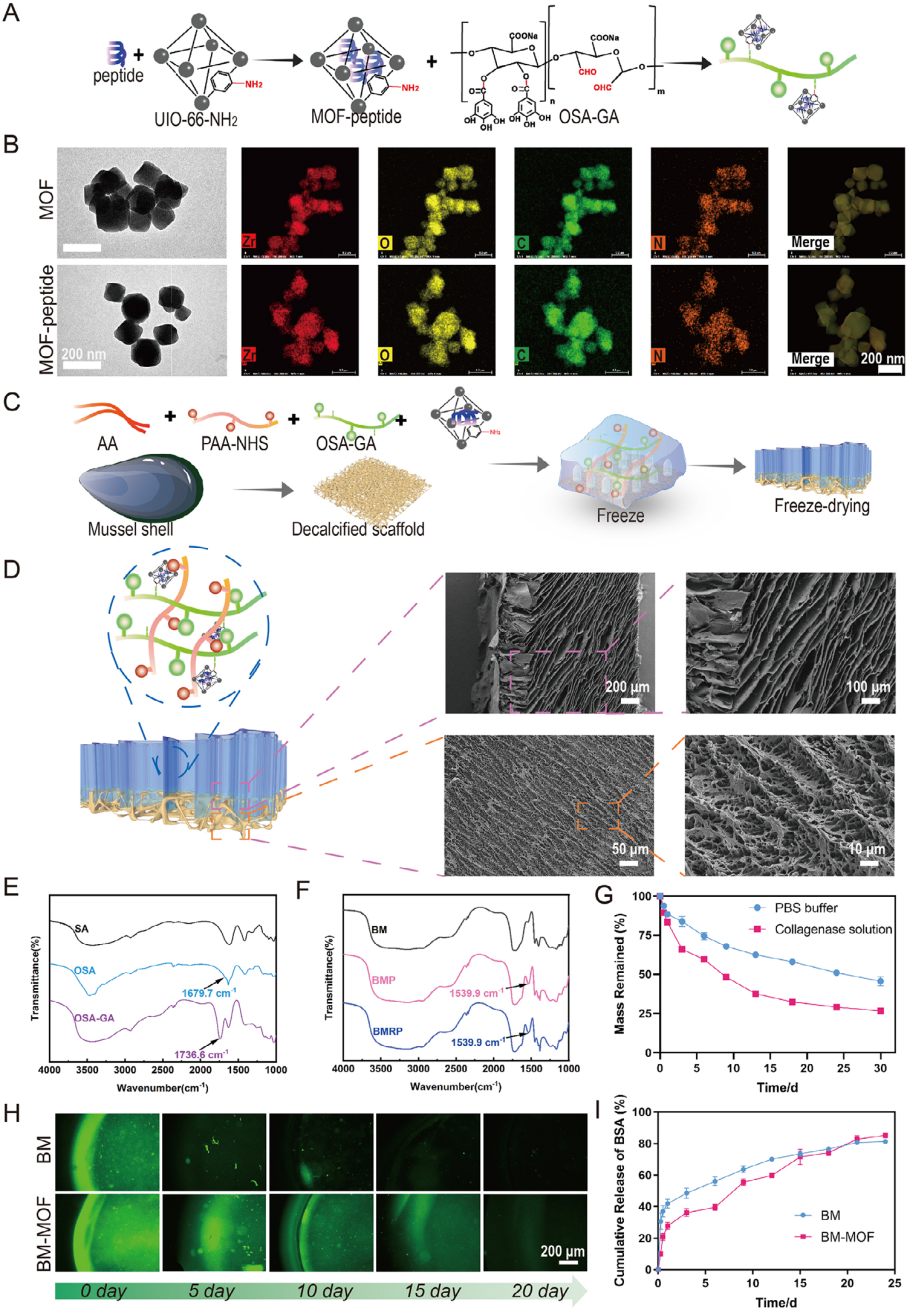
Characterization of MOF Loaded with Peptides and Biomimetic Microstructured Scaffolds. A) Schematic diagram of loading polypeptides into MOF and incorporating the MOF into the scaffold; B) TEM images of MOF and MOF‐peptide. Scale bar: 200 nm; C) Scheme of the construction of the biomimetic microstructured scaffold; D) SEM images of the biomimetic microstructured scaffold. Scale bar: 200, 100, 50, 10 µm; E,F) FTIR spectra of SA, OSA, OSA‐GA, BM, BMP, and BMRP scaffolds; G) Degradation rates of the scaffolds in PBS and collagenase solutions; H,I) Release profiles of the scaffolds in PBS at different time points, for BM scaffolds and the BM scaffolds containing MOF. Scale bar: 200 µm.

First, Teriparatide or R‐PTH was loaded into amino‐functionalized zirconium‐based metal–organic frameworks (UIO‐66‐NH_2_) (**Figure** **2**A). Metal‐organic frameworks (MOFs) have shown great potential in drug delivery due to their high porosity, large surface area, and tunable chemical properties. Particularly for short peptides, which are bioactive molecules, MOFs can provide a stable carrier that protects peptide drugs from in vivo enzymatic degradation and allows for controlled release.^[^ ^24^ ^]^ Transmission electron microscopy (TEM) imaging of the MOF and the peptide‐loaded MOF (MOF‐peptide) revealed that the pores of the MOF‐peptide were filled, confirming the effective loading of R‐PTH into the MOF. Energy‐dispersive X‐ray spectroscopy was used to observe the differences in elemental distribution between the MOF and MOF‐peptide (Figure 2B). In Figure 2C, a pre‐gel solution containing acrylic acid (AA), oxidized sodium alginate grafted with gallic acid (OSA‐GA), and sodium polyacrylate grafted with NHS (PAA‐NHS) was infused into a de‐mineralized oyster shell scaffold via the ice template method. Using N,N′‐methylenebisacrylamide (MBA) as the covalent crosslinker, multiple cross‐linking occurs through hydrogen bonds, ionic bonds, and a number of covalent and coordination bonds, leading to the preparation of a biomimetic microstructured scaffold (BM). Due to the presence of activated carboxyl and aldehyde groups, the scaffold can bind with the amino groups of the interfacing tissue, exhibiting excellent tissue adhesive properties (Figure S1, Supporting Information). Scanning electron microscopy (SEM) images of the BM scaffold microstructure (Figure 2D; Figure S2, Supporting Information) revealed that the gel layer prepared using the ice template method exhibited a directional pore structure,^[^ ^25^ ^]^ closely resembling the alignment of tendon fiber, which can provide directional guidance for the migration and climbing of tendon cells. Meanwhile, the demineralized mussel shell possesses a trabecular‐like microstructure, creating an ideal microenvironment for the growth and differentiation of osteoblasts.^[^ ^26^, ^27^ ^]^ In Figure 2E, a new characteristic peak at 1679.7 cm^−1^ appears in the infrared spectrum of OSA, attributed to the vibration of the aldehyde C═O, confirming the successful oxidation of SA. Additionally, compared to OSA, OSA‐GA shows a new absorption peak at 1736.6 cm^−1^, resulting from ester bond vibrations, indicating the formation of ester bonds between OSA and GA, thus confirming the effective grafting of GA onto OSA. In the DPPH scavenging assay, the BM scaffolds containing gallic acid are able to continuously scavenge free radicals (Figure S3, Supporting Information). In Figure 2F and Figure S4 (Supporting Information), the biomimetic tendon‐to‐bone microstructured scaffold (BMP) loaded with Teriparatide and the biomimetic tendon‐to‐bone microstructured scaffold (BMRP) loaded with R‐PTH both exhibit a deepening of the imine C═N stretching at 1539.9 cm^−1^, which results from the Schiff base reaction between the amino groups in UIO‐66‐NH2 and the aldehyde groups in OSA‐GA.

Next, the degradation performance of the BM scaffold was validated in vitro. In the PBS solution, the BM scaffold degraded by 50% by day 30, and the degradation rate of the BM scaffold was faster in PBS with added collagenase (Figure 2G). This may be due to the presence of some organic materials like collagen in the demineralized mussel shell scaffold. In Figure 2H,I, compared to simply mixing the drug into the BM scaffold, we found that the MOF enabled the BM scaffold to load more drugs with improved sustained release performance. This is likely because the drug‐loaded MOF stably binds to the BM scaffold through chemical bonds, achieving a slow‐release effect during the degradation of the scaffold. The degradation rate of scaffold materials must strike a balance among “mechanical support, degradation kinetics, and tissue regeneration.” In existing studies, natural materials (such as collagen, fibrin, chitosan, and hyaluronic acid) typically degrade via enzymatic hydrolysis (e.g., by collagenase) or hydrolysis, with relatively fast rates (ranging from weeks to months).^[^ ^28^, ^29^ ^]^ BMRP scaffold can provide sufficient mechanical (Figure S5, Supporting Information) support during the early stages to promote tissue repair while avoiding prolonged presence that could hinder new tissue growth or trigger foreign body reactions. The gradual degradation of the BMRP scaffold aligns with the sustained release of R‐PTH, ensuring extended therapeutic effects to balance bone homeostasis and remodel the microstructure.

### 2.2 Biomimetic Tendon‐to‐Bone Microstructured Scaffolds Repair Injured Cells, Regulate Immunity and Promote Cell Migration

**Figure 3:**
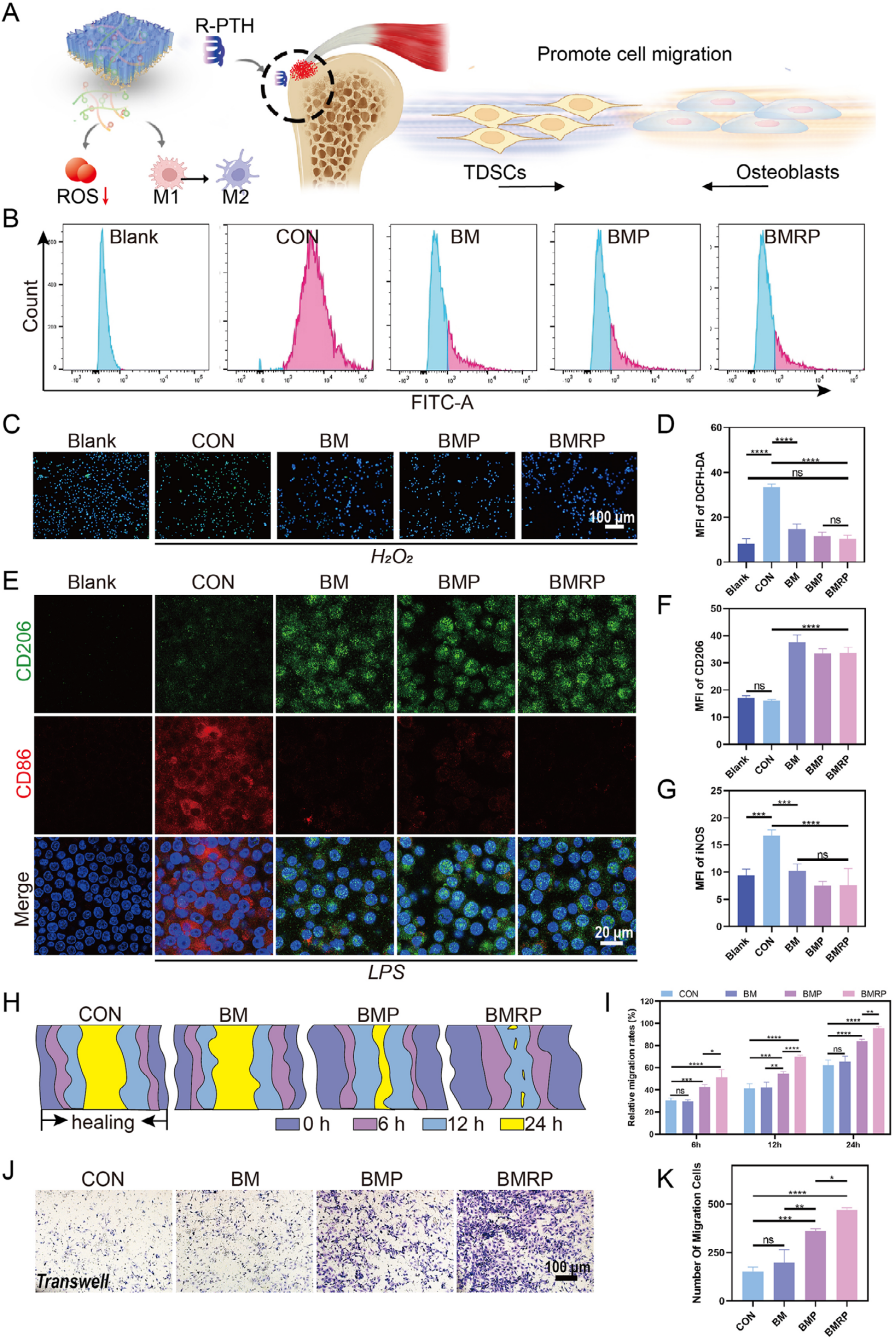
Antioxidant stress protection, immune modulation, and promotion of cell migration by the biomimetic microstructured scaffold loaded with R‐PTH. A) Schematic illustration of the multifunctional biomimetic microstructured scaffold; B) Flow cytometric analysis results for the clearance of intracellular ROS in MC3T3‐E1 cells under oxidative stress; C) Fluorescent images showing intracellular ROS clearance, with green indicating DCFH‐DA prob. Scale bar: 100 µm; D) Statistical analysis of intracellular DCFH‐DA fluorescence levels, (*n* = 4); E) Macrophage polarization experiment, with green indicating CD206 and red indicating CD86. Scale bar: 20 µm; F,G) Quantitative analysis of fluorescence intensity for CD206 and CD86, (*n* = 4); H) Scratch healing status at 0, 6, 12, and 24 h; I) Statistical analysis of Scratch healing at 6, 12, and 24 h, (*n* = 3); J) TDSCs migration through the transwell. Scale bar: 100 µm; K) Statistical analysis of the number of cells migrating through the Transwell, (*n* = 3). All data were presented as the mean ± SD, *ns P >* 0.05, *P < 0.05, **P < 0.01, ***P < 0.001, ****P < 0.0001.

Healing of the tendon‐to‐bone interface is a complex process involving the coordinated action of various cell types. Promoting the migration of osteoblasts and TDSCs plays a crucial role in this process.^[^ ^30^ ^]^ The biomimetic microstructured scaffold provides directional guidance for tendon cell migration, facilitating the integration of tendon cells with bone cells, which can significantly enhance the speed and quality of tendon‐to‐bone interface healing (**Figure** **3**A). Regulating the inflammatory microenvironment at the injured osteoporotic tendon‐to‐bone interface is particularly important.^[^ ^31^ ^]^ In the early stages of RCT, inflammatory cells (such as neutrophils and macrophages) accumulate in the interface microenvironment, leading to elevated local inflammatory factors and ROS, along with the upregulation of related enzymes (e.g., metalloproteinases). If the inflammation in the microenvironment is not actively controlled in a timely manner, it can result in an imbalance of inflammatory factors, further hindering healing of the tendon‐to‐bone interface and potentially leading to chronic inflammation. Destruction of the extracellular matrix in the microenvironment leads to bone loss, which further exacerbates osteoporosis, thereby entering an irreversible vicious cycle.^[^ ^32^ ^]^

First, CCK‐8 assays were conducted on biomimetic microstructured scaffolds containing different concentrations of MOF‐RPTH (0, 0.01, 0.1, 0.2, and 0.4 mg mL^−1^). The results showed that cell viability was affected to some extent when the concentration of MOF‐RPTH was 0.4 mg mL^−1^ (Figure S6, Supporting Information). For subsequent experiments, biomimetic microstructured scaffolds with a concentration of 0.2 mg mL^−1^ of MOF‐RPTH were selected. Live/dead staining and Phalloidin immunofluorescence staining experiments indicate that MC3T3‐E1 cells grow well on the scaffold materials (Figures S7, S8, Supporting Information). In the CCK‐8 assay, there were no significant differences in cell viability among the groups, demonstrating that BM, BMP, and BMRP scaffolds have good biocompatibility (Figure S9, Supporting Information). In vitro experiments, an oxidative stress cell injury model was constructed, and the injured cells were transferred to a biomimetic microstructured scaffold. It was found that different scaffolds effectively cleared ROS from the injured cells (Figure 3B,C). Compared to the control group, different biomimetic microstructured scaffold groups showed a statistically significant advantage (Figure 3D). This confirms that the BM, BMP, BMRP scaffolds have good restorative effects on oxidative stress injury in cells. This repair effect is primarily attributed to the gallic acid grafted onto the scaffold, which has excellent antioxidant and anti‐inflammatory properties, making it favored by researchers and widely applied in tissue engineering.^[^ ^33^ ^]^ Considering the excellent anti‐inflammatory and antioxidant properties of the biomimetic microstructured scaffold, its impact on macrophage polarization was examined. In Figure 3E, more iNOS‐labeled M1 macrophages were observed in the control group, while the different scaffold groups exhibited a higher presence of CD206‐labeled M2 macrophages. The fluorescence quantitative statistical analysis showed a statistical difference (Figure 3F,G).

In the scratch assay (Figure 3H,I; Figure S10, Supporting Information) the BMP and BMRP groups exhibited significantly enhanced TDSCs migration abilities at different time points. After 24 h, the scratches in the BMP and the BMRP groups were nearly closed, while the control group still showed noticeable scratches. In the transwell experiment (Figure 3J,K), the BMP and BMRP groups demonstrated a significantly stronger ability to migrate through the membrane compared to the BM group. It is worth noting that the BMRP group shows stronger promotion of cell migration compared to the BMP group, with statistically significant differences. Teriparatide activates the PTH1R receptor, initiating multiple signaling pathways that synergistically regulate cell migration.^[^ ^34^, ^35^ ^]^ First, the cAMP/PKA pathway promotes actin reorganization and pseudopod formation by inhibiting RhoA and activating Rac1/Cdc42. Second, the MAPK/ERK pathway upregulates the expression of matrix metalloproteinases (MMPs) and integrins, degrading the extracellular matrix (ECM) barrier and enhancing cell–matrix adhesion. Concurrently, the PLC‐IP3‐Ca^2^⁺/PKC pathway dynamically regulates cytoskeletal assembly and disassembly by phosphorylating focal adhesion‐associated proteins. Additionally, teriparatide clears ROS through the Nrf2/ARE pathway, maintaining cell viability, and recruits MSCs and osteoprogenitor cells to the injury site via paracrine secretion of chemokines such as vascular endothelial growth factor. These mechanisms collectively promote the efficient and directional migration of osteoblasts and tenocytes, providing critical impetus for bone repair and tendon‐to‐bone interface regeneration.^[^ ^36^, ^37^ ^]^ The BM scaffold loaded with our modified R‐PTH exhibits a stronger cell migration effect, which is highly beneficial for the integration of tendon cells with bone cells at the tendon‐to‐bone interface.

### 2.3 Biomimetic Tendon‐to‐Bone Microstructured Scaffolds Regulate Osteogenic and Tenogenic Differentiation

**Figure 4:**
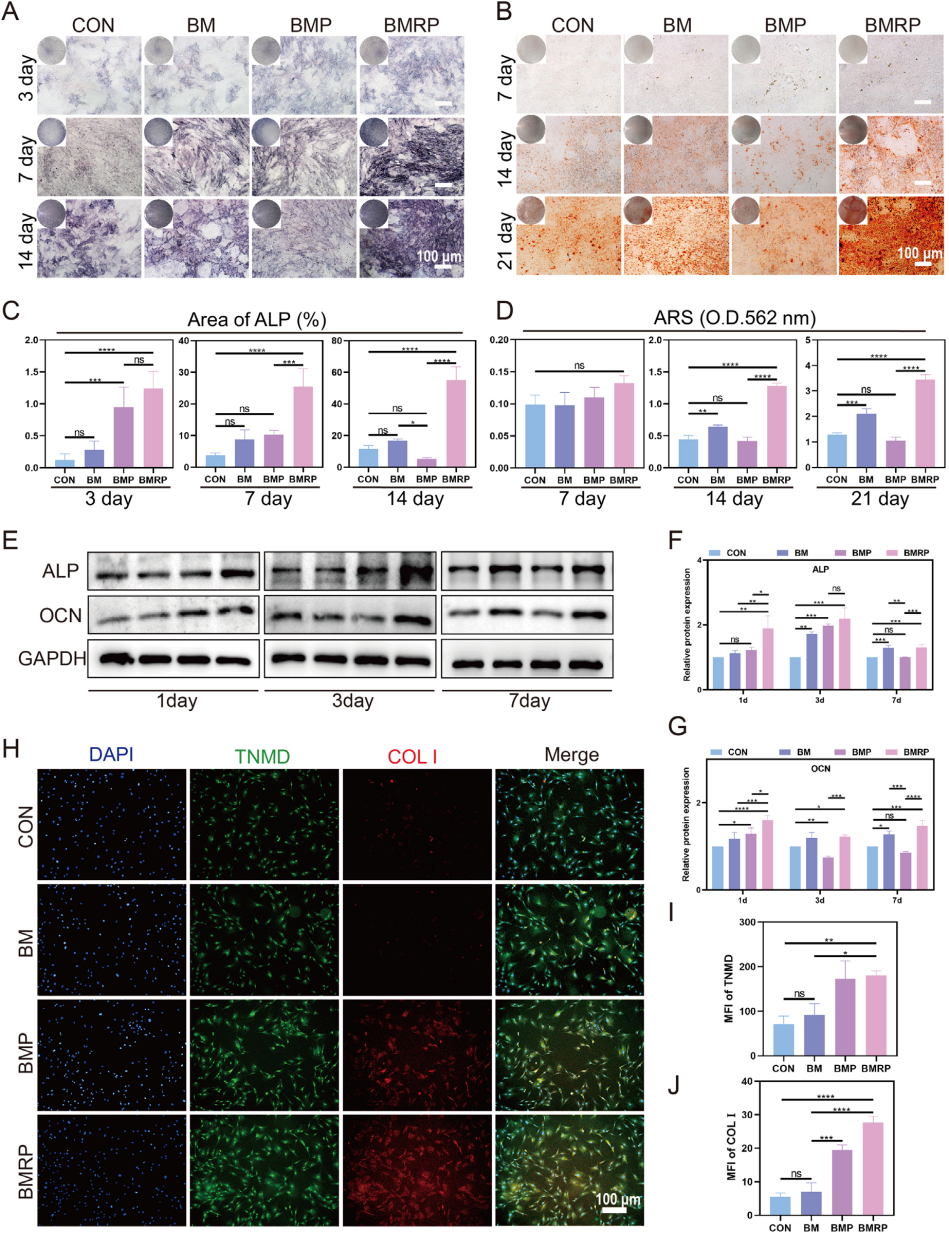
Biomimetic microstructured scaffolds loaded with R‐PTH promote osteogenic differentiation of BMSCs and tenogenic differentiation of TDSCs. A) ALP staining of BMSCs at 3, 7, and 14 days. Scale bar: 100 µm; B) ARS staining of BMSCs at 7, 14, and 21 days. Scale bar: 100 µm; C) Quantitative analysis of ALP staining at 3, 7, and 14 days, (*n* = 4); D) Quantitative analysis of ARS staining at 7, 14, and 21 days, (*n* = 4); E) Western blot analysis of osteogenesis‐related proteins ALP and OCN in BMSCs at 1, 3, and 7 days; Quantitative analysis of ALP (F) and OCN (G) expression levels in BMSCs at 1, 3, and 7 days, (*n* = 3); H) Immunofluorescence of TNMD (green) and COL I (red) in TDSCs after 3 days of culture. Scale bar:100 µm; Quantitative analysis of TNMD (I) and COL I (J) expression, (*n* = 3). All data were presented as the mean ± SD, *ns P >* 0.05, *P < 0.05, **P < 0.01, ***P < 0.001, ****P < 0.0001.

Due to osteoporotic RCT, there is abnormal bone metabolism, bone loss, and destruction of the bone microstructure at the tendon‐to‐bone interface. Thus, how to efficiently promote osteogenesis is crucial for the healing of the tendon‐to‐bone interface.^[^ ^2^, ^38^ ^]^ Alkaline phosphatase (ALP) staining and Alizarin red staining (ARS) were conducted at different time points to assess the osteogenic mineralization ability of different scaffolds (**Figure** **4**A,B). On day 3, the BMP and BMRP groups exhibited stronger ALP expression, showing a significant statistical difference compared to the control group (Figure 4C). On days 7 and 14, there was no significant difference in ALP expression between the BMP group and the control group, while the BMRP group consistently displayed significantly higher ALP expression than the other groups. On day 7, ARS staining results did not show significant differences among the groups. However, on days 14 and 21, the BMRP group had significantly more mineralized calcium nodules than the other groups, whereas the BMP group did not show a significant difference compared to the control group (Figure 4D). Notably, the BM group demonstrated some osteogenic activity, with more calcium nodules formed by the BM group compared to the control group on days 14 and 21.

Subsequently, an investigation into the effects of the scaffolds on the expression of osteogenic‐related proteins was conducted at various time points (Figure 4E; Figure S11, Supporting Information). Osteocalcin (OCN) plays a crucial role in the osteogenic process, not only promoting osteoblast mineralization and activity but also regulating bone metabolism and participating in hormone‐signaling pathways to support bone health and strength.^[^ ^39^ ^]^ On days 1, 3, and 7, the BMRP group showed a significant upregulation in the expression of ALP and OCN, while the BMP group exhibited a marked inhibitory effect on OCN expression (Figure 4F,G). Additionally, the BM group showed a certain upregulation of OCN expression on days 3 and 7, which may be attributed to the osteogenic activity of the demineralized mussel shell in the BM scaffold.^[^ ^40^ ^]^ The inhibitory effect on osteogenic differentiation observed in the BMP group further confirms that Teriparatide exhibits a significant reduction in its osteogenic effects under continuous stimulation. The BMP scaffold continuously releases Teriparatide, leading to prolonged activation of the PTH receptor. This sustained activation can result in receptor internalization or downregulation, weakening pro‐osteogenic signaling pathways such as the cAMP/PKA pathway. Additionally, continuous stimulation may inhibit osteogenesis‐related pathways like Wnt/β‐catenin or induce negative feedback factors (such as SOST), thereby limiting osteoblast differentiation.^[^ ^36^, ^37^ ^]^ In contrast, the BMRP scaffold does not cause similar osteogenic inhibition. R‐PTH selectively activates only the PKC pathway and does not rely on PLC or cAMP/PKA. R‐PTH enhances β‐catenin stability through a PKC‐dependent mechanism by inhibiting degradation and promoting synthesis. Then, tendon stem cells were seeded on the scaffold and cultured for three days. Col I and Tenomodulin (TNMD), as tendon‐specific markers, were examined by immunofluorescence staining (Figure 4H). It was found that both the BMP group and the BMRP group significantly enhanced the expression of Col I and TNMD (Figure 4I,J).

### 2.4 Biomimetic Tendon‐to‐Bone Microstructured Scaffolds Regulate the Balance between Bone Formation and Bone Resorption

**Figure 5:**
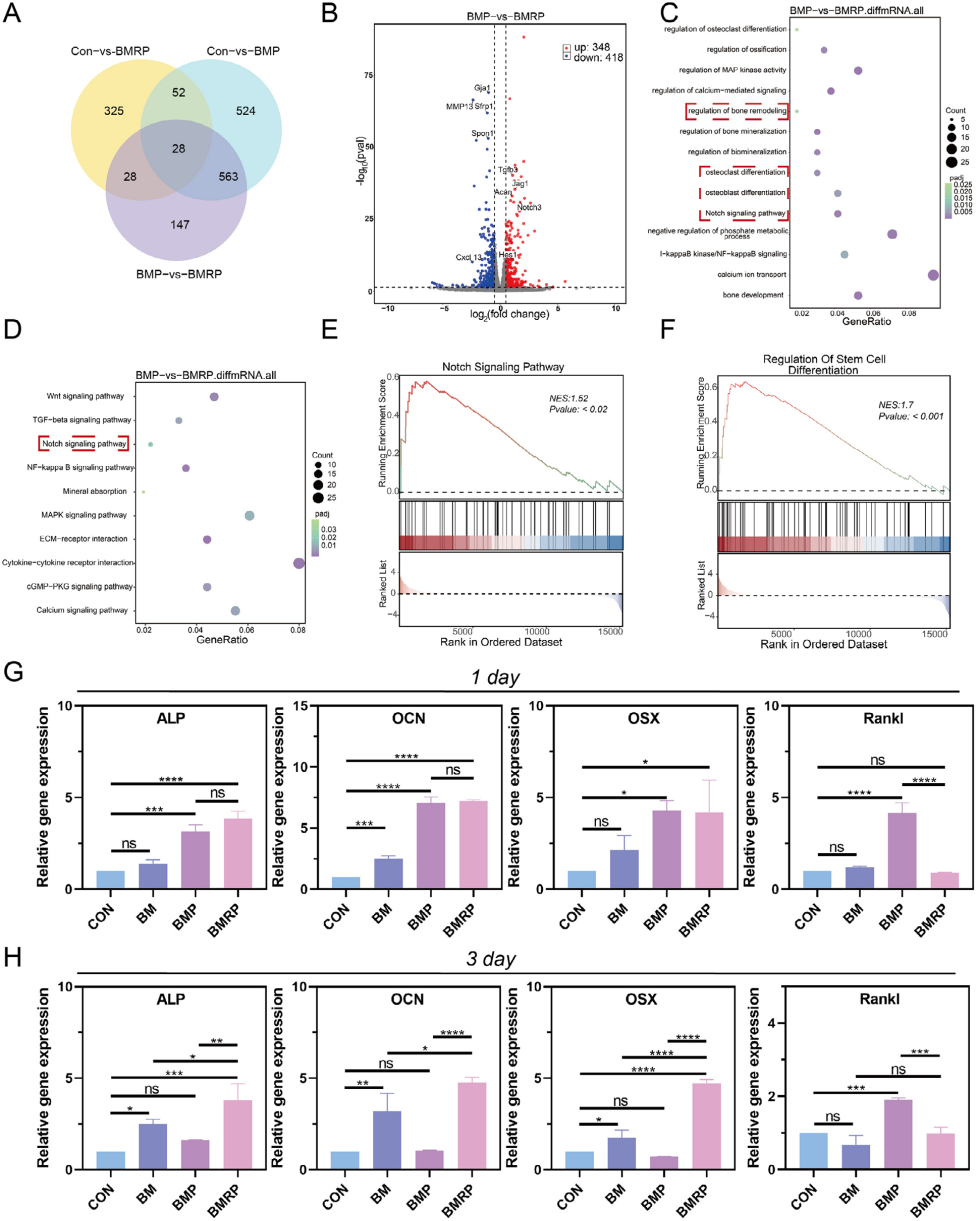
RNA sequencing analysis and related gene expression in BMSCs. A) Venn diagram of DEGs between CON versus BMRP, CON versus BMP, and BMP versus BMRP; B) Volcano plot of gene expression analysis by RNA‐seq for BMP and BMRP groups; C) Top 10 pathways enriched by DEGs; D) GO terms associated with bone metabolic processes; E,F) GSEA plots illustrating the enrichment of DEGs in Notch signaling pathway and regulation of stem cell differentiation (*p*‐value < 0.05); G) Statistical analysis of Gene expression levels of ALP, OCN, OSX, and RANKL on day 1, (*n* = 3); H) Statistical analysis of Gene expression levels of ALP, OCN, OSX, and RANKL on day 3, (*n* = 3). All data were presented as the mean ± SD, *ns P >* 0.05, *P < 0.05, **P < 0.01, ***P < 0.001, ****P < 0.0001.

BMSCs were seeded on different scaffolds and cultured for three days, after which the BMSCs were collected, and RNA was isolated for transcriptome sequencing (RNA‐seq) analysis. Principal component analysis revealed significant differences in RNA expression profiles among the groups. There were 766 differentially expressed genes (DEGs) between the BMP and BMRP groups, including 348 upregulated mRNAs and 418 downregulated mRNAs (**Figure** **5**AB). An in‐depth analysis of the DEGs involved in bone metabolism, including Gja1, MMP13, SFRP1, SPON1, Cxcl13, TGFB3, JAG1, ACAN, NOTCH3, and HES1, was carried out. Based on these differences, gene ontology (GO) analysis was performed to identify the biological functions of the proteins encoded by the differentially expressed mRNAs (Figure 5C). The DEGs between the two groups were primarily enriched in biological processes, including calcium ion transport (GO:0 006816), regulation of calcium‐mediated signaling (GO:00 50848), regulation of MAP kinase activity (GO:00 43405), regulation of bone mineralization (GO:00 30500), Notch signaling pathway (GO:0 007219), regulation of biomineralization (GO:011 0149), and osteoclast differentiation (GO:00 30316). Kyoto encyclopedia of genes and genomes (KEGG) enrichment analysis further identified pathways associated with the DEGs, as shown in Figure 5D. Compared to the BMP group, the BMRP group exhibited enrichment of DEGs in bone metabolism‐related pathways, including the Wnt signaling pathway, TGFβ signaling pathway, and Notch signaling pathway, all of which regulate osteogenesis and bone resorption.^[^ ^41^, ^42^, ^43^, ^44^ ^]^ Gene set enrichment analysis (GSEA) indicated that the Notch signaling pathway (NES = 1.52, P = 0.02) and regulation of stem cell differentiation (NES = 1.7, *P* < 0.001) were negatively enriched in the BMP group, suggesting that the BMP scaffold inhibited the activation of the Notch signaling pathway and regulation of stem cell differentiation (Figure 5E,F).

RNA‐seq analysis results indicated that the BMP and BMRP groups regulated the biological processes of BMSCs through various mechanisms, with the main differences lying in bone mineralization and bone resorption. Real‐time quantitative polymerase chain reaction (RT‐qPCR) was used to study the expression levels of osteogenic‐related genes at different time points, exploring the impact of different scaffolds on BMSCs' osteogenic differentiation. In Figure 5G,H, on day 1, both the BMP and BMRP groups significantly upregulated the gene expression levels of ALP, OCN, and Osterix 1 (OSX) in BMSCs. By day 3, the BMP group exhibited some inhibitory effects on osteogenic‐related genes, while the BMRP group consistently showed strong osteogenic activity. Compared to the control group, the BM scaffold upregulated osteogenic‐related genes. Bone resorption is primarily dominated by osteoclasts, and BMSCs influence the differentiation, maturation, and resorptive functions of osteoclasts through complex direct and indirect mechanisms.^[^ ^45^ ^]^ This interaction is crucial for maintaining normal bone metabolism and repair processes. BMSCs can secrete various cytokines and growth factors, such as RANKL (receptor activator of nuclear factor κB ligand), which play a key role in regulating osteoclast differentiation and activity.^[^ ^45^, ^46^ ^]^ In Figure 5G,H, it can be observed that on days 1 and 3, the BMP scaffold promoted RANKL gene expression. However, the RANKL expression levels in the BM and BMRP groups were similar to those in the control group. Therefore, the BMP scaffold and the BMRP scaffold influence the bone metabolism balance at the tendon‐to‐bone interface through the mediation of BMSCs.

**Figure 6:**
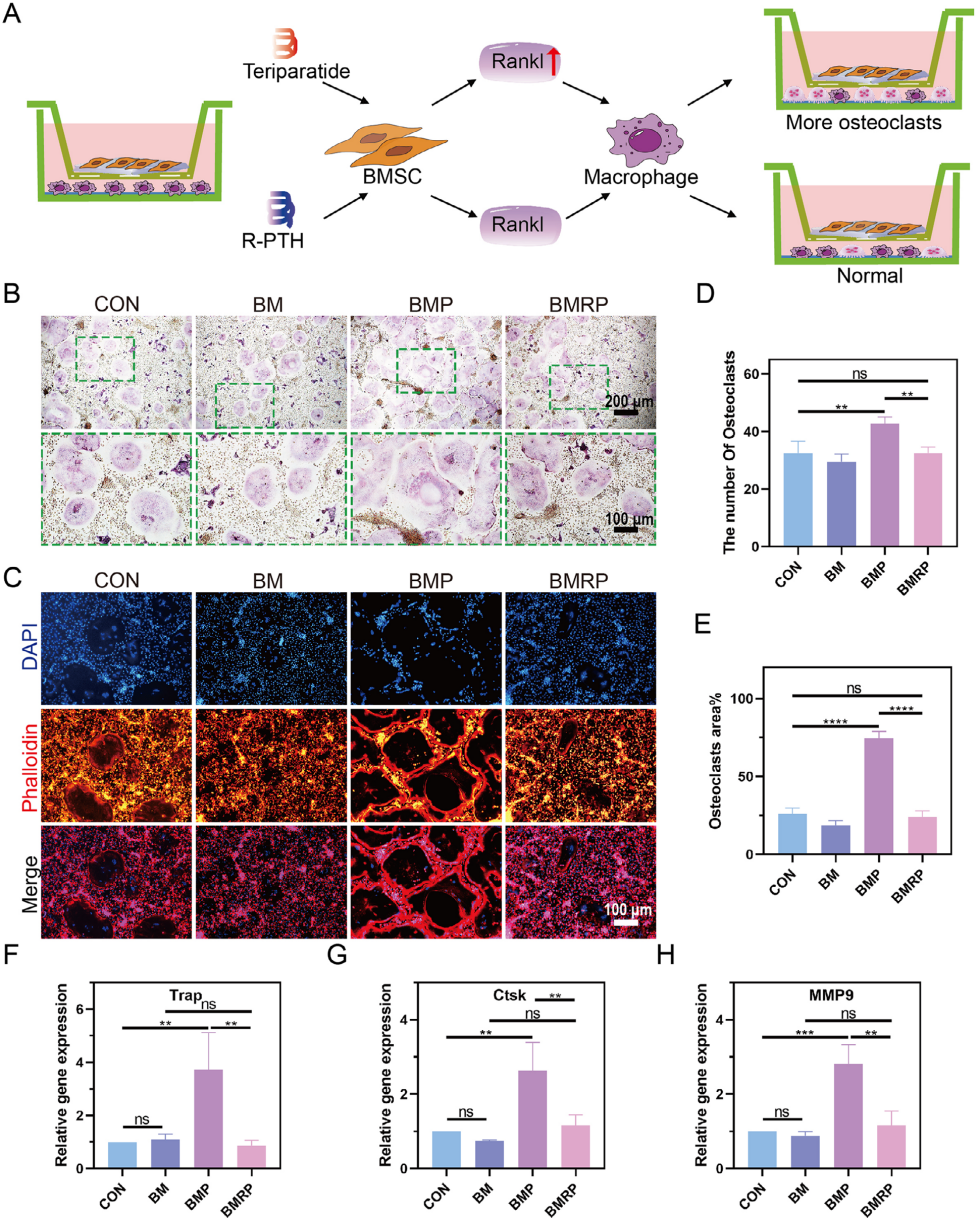
The effect of BMSCs seeded on the scaffolds on osteoclast differentiation. A) Schematic illustration of the co‐culture system of BMSCs and macrophages; B) After co‐culture for 3 days, TRAP staining of macrophage differentiation into osteoclasts. Scale bar: 200 µm,100 µm; C) Immunofluorescence staining of the cell cytoskeleton after three days of co‐culture, where red represents phalloidin and blue represents DAPI. Scale bar: 100 µm; D) Quantitative analysis of the number of osteoclasts, (*n* = 4); E) Quantitative analysis of the area of osteoclasts, (*n* = 4); F–H) represent the quantitative analysis of the expression levels of TRAP, Ctsk, and MMP9 genes in osteoclasts, respectively, (*n* = 3). All data were presented as the mean ± SD, *ns P >* 0.05, **P < 0.01, ***P < 0.001, ****P < 0.0001.

Osteoclasts play a vital role in the repair, regeneration, and microstructural remodeling of the tendon‐to‐bone interface. They precisely regulate the balance between bone resorption and remodeling, rebuilding the biomechanical properties and structural integrity at the tendon‐to‐bone junction.^[^ ^47^ ^]^ For the specific location of the osteoporotic tendon‐to‐bone interface, inhibiting osteoclasts or excessively activating them is detrimental to the repair of the tendon‐to‐bone interface.^[^ ^6^ ^]^ When bone marrow‐derived macrophages were cultured on different scaffold materials, the different scaffolds did not affect osteoclast differentiation (Figure S12, Supporting Information). Combining the previous RNA‐seq analysis and RT‐qPCR results, it appears that different scaffolds may influence osteoclast differentiation through BMSCs. Co‐culture experiments involving macrophages and BMSCs were conducted to investigate osteoclast differentiation (**Figure** **6**A). Following the induction of culture, tartrate‐resistant acid phosphatase (TRAP) staining was performed (Figure 6B). As shown in Figure 6C, the BMP group had a significantly higher number of osteoclasts compared to the other groups, demonstrating statistical differences. In Figure 6D,E, staining with phalloidin revealed that the BMP group formed larger osteoclasts and sealing zones. The formation of excessive osteoclasts and larger sealing zones indicates that osteoclasts can concentrate their resorptive functions in specific areas, thereby disrupting the balance between bone formation and resorption. In the BM and BMRP groups, osteoclasts showed no significant differences in quantity or size compared to the control group, indicating that the BMRP scaffold does not affect the normal differentiation of osteoclasts.

Osteoclasts rely on various enzymes to perform their functions during bone resorption, primarily including Cathepsin K (Ctsk), Matrix Metalloproteinase 9 (MMP9), and TRAP. Cathepsin K plays a major role in the bone resorption activities of osteoclasts by degrading collagen in the bone matrix. MMP9 is capable of degrading various ECM components and participates in the degradation of non‐collagenous components.^[^ ^48^, ^49^ ^]^ Through RT‐qPCR experiments, it was found that the gene expression of osteoclast‐related enzymes in the BMP group was significantly higher than in the other groups, while the expression levels in the BMRP group were similar to those in the control group (Figure 6F–H). This indicates that the osteoclasts in the BMP group were overactivated, exhibiting stronger bone resorption capabilities, whereas the BMRP group did not affect the activity of osteoclasts in the normal tendon‐to‐bone interface microenvironment. The BMRP scaffold showed a greater advantage over the BMP scaffold in regulating the balance between bone formation and resorption. This is primarily because R‐PTH selectively activates the PKC signaling pathway without triggering the cAMP/PKA and PLC pathways. This selective activation promotes osteogenic differentiation via increased β‐catenin levels (by inhibiting degradation and promoting synthesis), leading to enhanced expression of osteogenic markers (such as ALP and OCN) and mineralized nodule formation, while avoiding the upregulation of RANKL and subsequent osteoclast activation.^[^ ^23^ ^]^

### 2.5 Biomimetic Tendon‐to‐Bone Microstructured Scaffolds Promote Repair of Osteoporotic RCT

**Figure 7:**
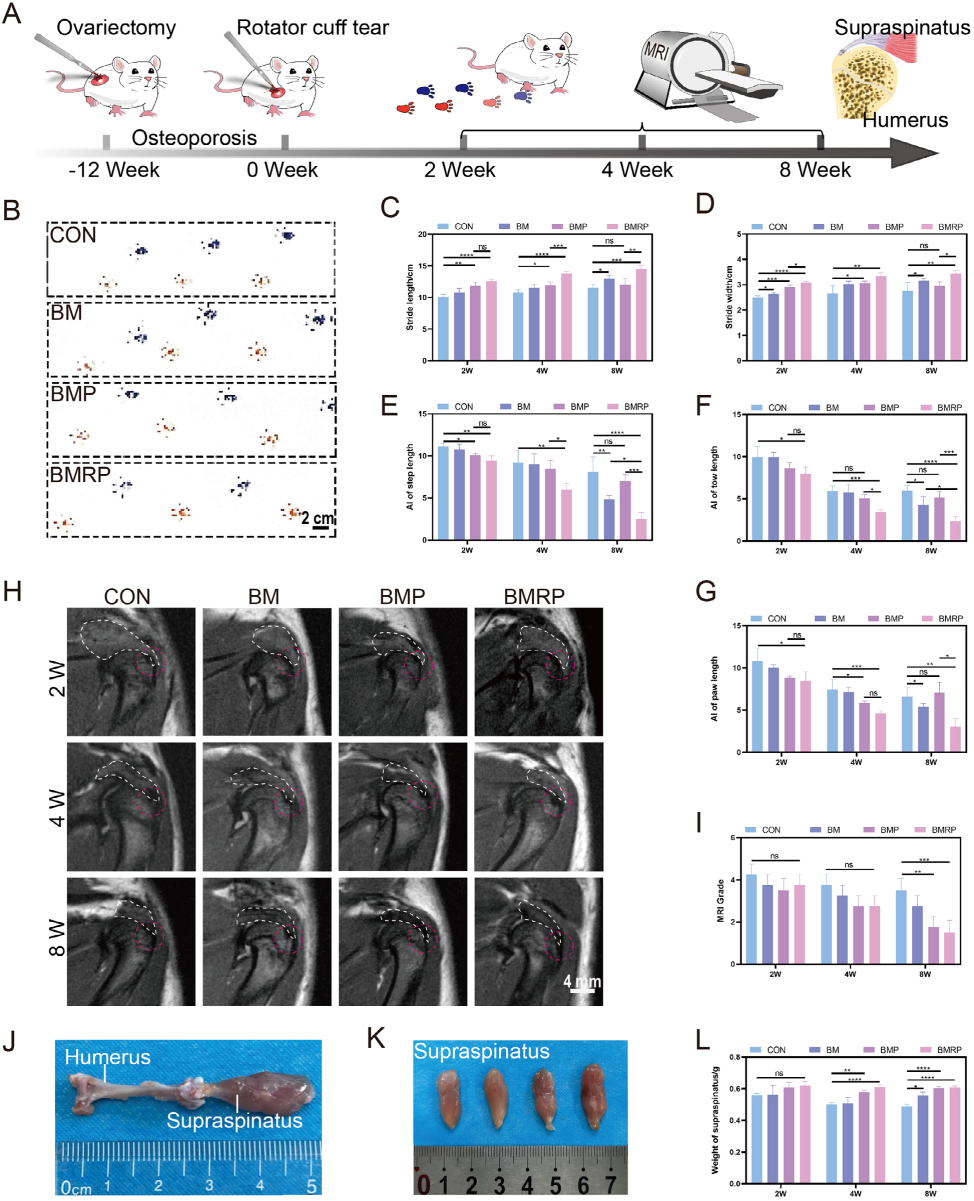
Functional reconstruction of osteoporotic RCT using a biomimetic microstructured scaffold loaded with R‐PTH. A) Schematic illustration of the model construction and functional assessment of osteoporotic RCT; B) Footprint photographs 8 weeks post‐surgery for rotator cuff repair. Scale bar:2 cm; C,D) Statistical analysis of stride length and stride width parameters at 2, 4, and 8 weeks, (*n* = 4); E) Stride length, F) paw length, and G) toe spread asymmetry index statistical analysis, (*n* = 4); H) MRI images of the shoulder joint at 2, 4, and 8 weeks. Scale bar: 4 mm; I) MRI evaluation grades for the rotator cuff, (*n* = 4); J) Photograph of the humerus and supraspinatus; K) Photograph of the supraspinatus muscle at 8 weeks; L) Statistical analysis of supraspinatus mass at 2, 4, and 8 weeks, (*n* = 4). All data were presented as the mean ± SD, *ns P >* 0.05, *P < 0.05, **P < 0.01, ***P < 0.001, ****P < 0.0001.

First, ovariectomy was performed on female Sprague–Dawley (SD) rats, and micro‐CT scans of the femur were conducted at week 12 to confirm the successful establishment of the osteoporosis model (Figures S13, S14, Supporting Information). At week 12, RCR surgery was performed, and different biomimetic microstructured scaffolds were implanted (Figure S15, Supporting Information). Subsequently, MRI was used to observe changes in the supraspinatus after injury, while gait analysis and supraspinatus weight assessment were conducted to evaluate the repair effects of different biomimetic microstructured scaffolds on the healing of the tendon‐to‐bone interface (**Figure** **7**A). At different time points (weeks 2, 4, and 8), gait imprint analysis was performed on the SD rats in different groups. Red imprints indicated the surgical side, while blue imprints indicated the normal side (Figure 7B; Figures S16, S17, Supporting Information). At week 2, the BMP and BMRP groups showed some recovery in stride length and stride width compared to the control group (Figure 7C–E). The paw parameters (paw length and paw spread) showed a slight recovery in the BMRP group (Figure 7F,G). At week 4, the BMRP group showed significant advantages in the recovery of stride length, stride width, and paw parameters. By week 8, all groups exhibited varying degrees of recovery in gait parameters. Notably, the BMP group did not show advantages in the later gait parameters. Compared to the control group, the BM group exhibited some advantages. The BMRP group showed significant recovery across all parameters, indicating that BMRP provided the best repair effects and promoted functional recovery of the osteoporotic tendon‐to‐bone interface.

Magnetic resonance imaging (MRI) of the shoulder is a necessary examination for clinically diagnosing RCT and can also observe the recovery status after RCR. MRI of the shoulders of osteoporotic rats at different time points can effectively monitor the dynamic repair process of the tendon‐to‐bone interface (Figure 7H). At week 2, edema was observed in the supraspinatus of all groups, with the control group showing the most significant edema. By week 4, the control group exhibited some degree of atrophy in the supraspinatus, and mild edema was noted at the tendon‐to‐bone interface. Compared to the BM group, the BMP and BMRP groups demonstrated better morphological recovery of the supraspinatus, although no significant statistical differences were found among the groups in MRI grading (Figure 7I, Table S1, Supporting Information). At week 8, the control group showed obvious atrophy and fatty degeneration of the supraspinatus, while the BM group exhibited relatively mild changes. The BMP and BMRP groups restored the supraspinatus to normal signal intensity, with the tendon end showing normal tendon signals, indicating significant advantages in MRI grading with statistical differences. At different time points, the weight of the supraspinatus was measured for comparison (Figure 7J,K). After RCR, the control group demonstrated a decreasing trend in muscle mass, while the other groups showed no significant changes (Figure 7L). The tendon ends of the supraspinatus in the BMP and BMRP groups were similar to those in normal tendon areas, with no signs of tendon degeneration.

Therefore, based on the above results, the BMRP group showed significant advantages in functional recovery of the rotator cuff and in preventing atrophy and degeneration of the supraspinatus. Notably, both the BMP and BMRP groups were able to prevent atrophy and degeneration of the injured supraspinatus. Skeletal muscle is a target organ for various hormones, such as insulin and estrogen.^[^ ^50^ ^]^ PTH may be one of these hormones mediating its effects, which is different from its traditional role as a regulator of mineral homeostasis.^[^ ^51^ ^]^ Recent studies have reported that Teriparatide, by inhibiting lipid droplet accumulation and promoting muscle regeneration and the expression of beige fat cells, reversibly improves muscle quality and atrophy following chronic RCT.^[^ ^52^ ^]^ Compared to Teriparatide, the BMRP group, which continuously releases our prepared R‐PTH, exhibited better repair effects.

### 2.6 Biomimetic Tendon‐to‐Bone Microstructured Scaffolds Regulate Bone Homeostasis of Osteoporotic Tendon‐to‐Bone Interface

**Figure 8:**
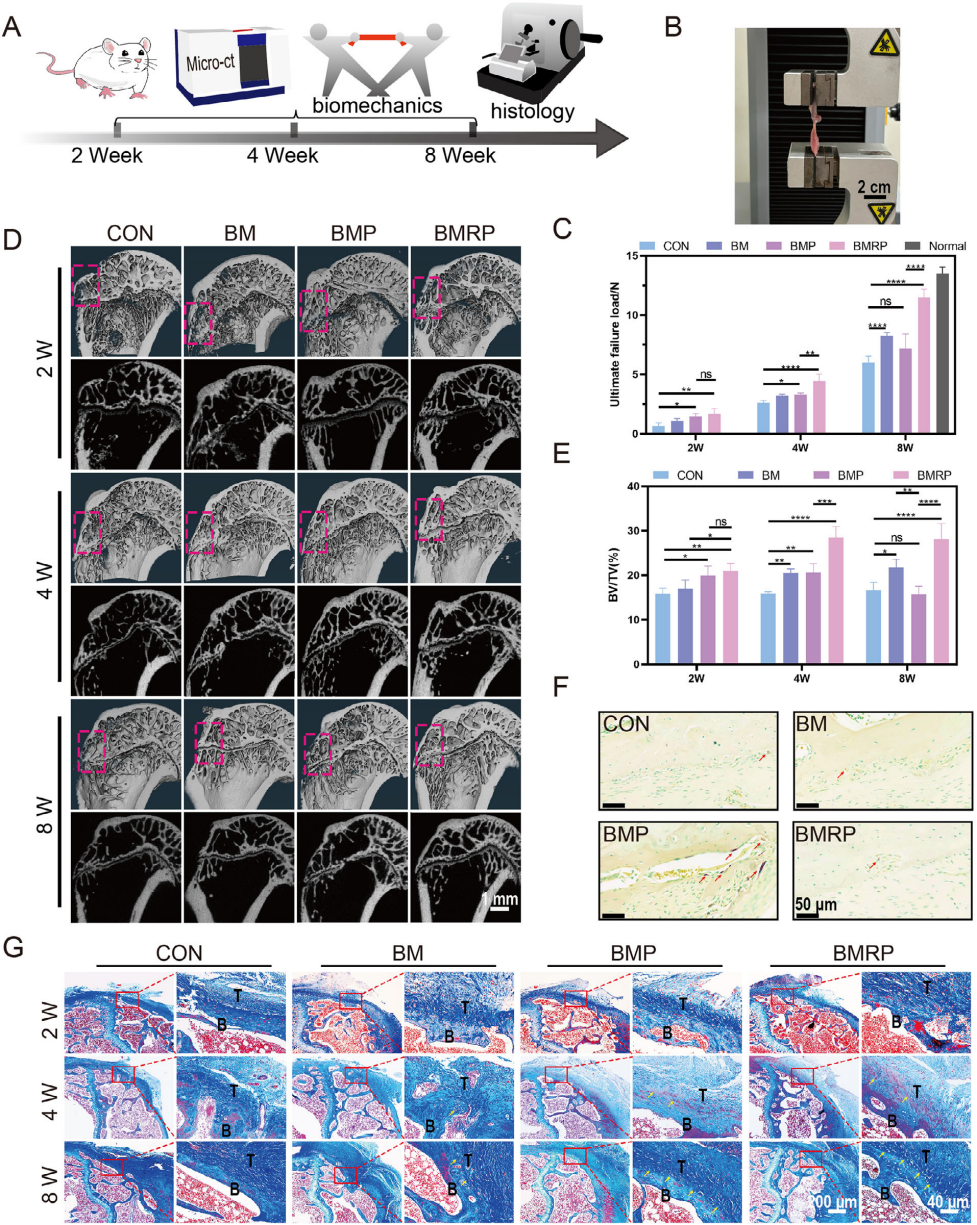
Biomechanical and bone microstructure remodeling of the osteoporotic tendon‐to‐bone interface. A) Schematic illustration of the biomechanical, micro‐CT, and histological analyses performed at 2, 4, and 8 weeks post‐surgery for osteoporotic RCR; B) Photographs of the biomechanical testing. Scale bar:2 cm; C) Statistical analysis of the maximum load that the osteoporotic tendon‐to‐bone interface can withstand, (*n* = 4); D) Micro‐CT 3D reconstruction and 2D coronal plane images of the humerus at 2, 4, and 8 weeks. Scale bar: 1 mm; E) Quantitative analysis of BV/TV at the injury site of the tendon‐to‐bone interface, (*n* = 4); F) TRAP staining of the tendon‐to‐bone interface at 8 weeks; G) Masson staining of the tendon‐to‐bone interface at 2, 4, and 8 weeks. B: bone, T: tendon, yellow arrows: Sharpey fibers. Scale bar: 200 µm,40 µm. All data were presented as the mean ± SD, *ns P >* 0.05, *P < 0.05, **P < 0.01, ***P < 0.001, ****P < 0.0001.

At different time points after RCR in osteoporotic rats, biomechanical experiments were conducted to assess the integration strength between the supraspinatus and bone. Changes in bone quality at the tendon‐to‐bone interface were observed using micro‐CT, followed by histological analysis of the balance between Bone formation and bone resorption (**Figure** **8**A). The ends of the humerus and supraspinatus were fixed in a universal testing machine to measure the ultimate failure load that the tendon‐to‐bone interface could withstand (Figure 8B). Over time, the maximum load that each group could bear gradually increased (Figure 8C). In the early stages of injury repair, both the BMP and BMRP groups could withstand greater tensile forces. However, in the later stages of repair, no significant differences were observed between the BMP group and the control group. By week 8, the BM group could withstand greater tensile forces than both the control and BMP groups. The maximum tensile force that the BMRP group could withstand was similar to that of the normal group. In Figure 8D, it displays the 3D reconstruction images from micro‐CT, along with the 2D coronal images of the humeral. At week 2, compared to the control group, the BMP and BMRP groups showed significantly greater bone quality at the tendon‐to‐bone interface, with statistical differences (Figure 8E). By week 4, the BM group exhibited bone quality similar to that of the BMP group, while the BMRP group showed markedly higher bone quality than the other groups. Notably, at week 8, the BMP group experienced a decrease in bone quality, which aligns with previous cellular osteogenic experimental results, possibly due to the continuous release of Teriparatide at the tendon‐to‐bone interface by the BMP scaffold. The BMRP group, which released R‐PTH, continuously acted on the osteoporotic tendon‐to‐bone interface, enhancing bone quality at the interface. By week 8, the BMP group showed a greater number of osteoclasts compared to the other groups, indicating that under the continuous influence of Teriparatide, bone resorption at the tendon‐to‐bone interface significantly increased (Figure 8F).

Through Masson staining (Figure 8G), the remodeling process of the interface microstructure can be observed. At week 2, no fusion was observed between the tendon and bone in any group, although new bone formation was visible in the BMP and BMRP groups. By week 4, the tendon fibers in the control group exhibited disorganized arrangement, while the BM group showed partial fusion between the tendon and bone. The BMP and BMRP groups displayed newly formed microstructures with gradient mineralization, consisting of new bone, mineralized fibrocartilage, and tendon fibers. The arrangement of new collagen fibers was also more organized, with an increase in the formation of Sharpey fibers, indicating the establishment of new tendon insertion points at the bone surface. At week 8, the tendon‐to‐bone interface in the control group showed simple healing and did not exhibit new microstructural formation compared to the other groups. The BM group displayed fusion between bone and tendon. Remarkably, the BMP and BMRP groups formed a mature enthesis, consisting of a four‐layer microstructure composed of mineralized bone, mineralized cartilage, fibrocartilage, and tendon. However, compared to the BMRP group, the mineralized bone in the BMP group was thinner, indicating insufficient depth of tendon insertion into the bone, which explained the lack of significant improvements in biomechanical performance in the BMP group during the later stages of repair.

Overall, Teriparatide activates various intracellular signaling pathways, such as cAMP‐PKA, MAPK/ERK, and PI3K/Akt, by binding to PTH/PTHrP receptors on target cells, thereby influencing cellular functions and metabolism.^[^ ^53^ ^]^ In the early stages of repair at the osteoporotic tendon‐to‐bone interface, Teriparatide promoted interface healing while inhibiting atrophy and fatty degeneration of the supraspinatus muscle. However, due to the unique characteristics of the osteoporotic tendon‐to‐bone interface, the prolonged action of Teriparatide can excessively activate osteoclasts, disrupting the bone microstructure, leading to reduced biomechanical properties of the enthesis. The BMRP group, which released R‐PTH, promoted osteogenesis without affecting the remodeling of the interface by osteoclasts. The BMRP group reconstructed the microstructure through a biomimetic microstructured scaffold, balancing the bone homeostasis of the osteoporotic tendon‐to‐bone interface, allowing the soft tendon tissue to better integrate with the hard bone tissue.

### 2.7 Biomimetic Tendon‐to‐Bone Microstructured Scaffolds Regulate Immune Responses and Collagen Neogenesis

**Figure 9:**
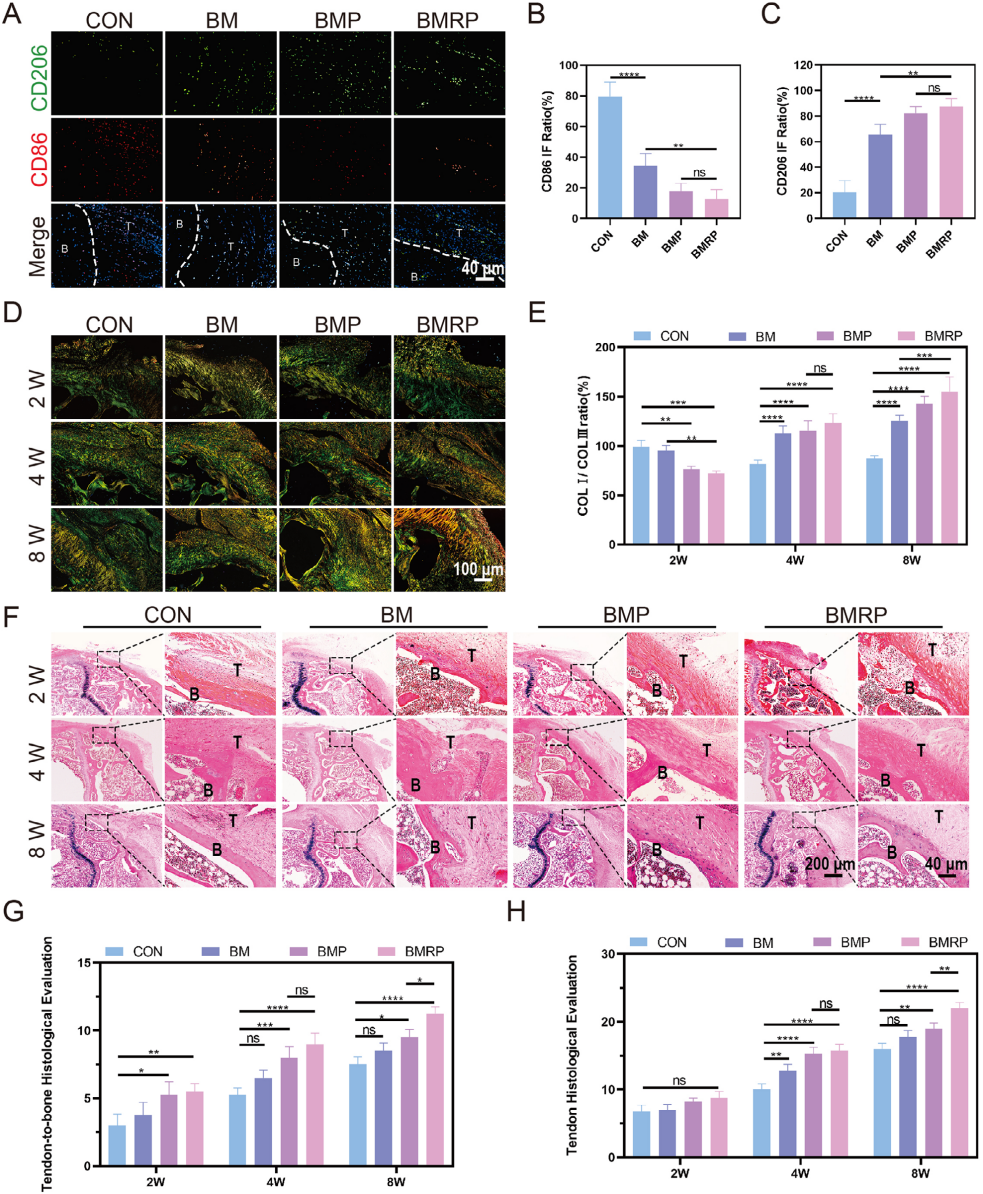
Regulation of interface inflammatory response and promotion of collagen regeneration by a biomimetic microstructured scaffold loaded with R‐PTH. A) Immunofluorescence staining of CD206 (green) and CD86 (red) at the osteoporotic tendon‐to‐bone interface at 2 weeks. Scale bar: 40 µm; B,C) Fluorescence quantification of CD86, CD206, (*n* = 4); D) Sirius Red staining at 2, 4, and 8 weeks, with red indicating COL I and green indicating COL III. Scale bar: 100 µm; E) Quantitative analysis of COL I/COL III ratio at 2, 4, and 8 weeks, (*n* = 4); F) HE staining of the tendon‐to‐bone interface at 2, 4, and 8 weeks. B: bone, T: tendon, Scale bar: 200 µm, 40 µm; G) Histological scoring of the tendon‐to‐bone interface at 2, 4, and 8 weeks, (*n* = 4); H) Histological scoring of tendon tissue at 2, 4, and 8 weeks, (*n* = 4). All data were presented as the mean ± SD, *ns P >* 0.05, *P < 0.05, **P < 0.01, ***P < 0.001, ****P < 0.0001.

Immunofluorescence staining was performed at injured sites to analyze the immunophenotypic profile of the tendon‐to‐bone interface microenvironment. M2 macrophages were selectively labeled with CD206, while M1 macrophages were identified via CD86 immunostaining, enabling differential characterization of macrophage polarization states (**Figure** **9**A). The control group exhibited a higher presence of M1 macrophages, while the other groups showed more M2 macrophages (Figure 9B,C). Compared to the control group, the different scaffold groups displayed less aggregation of inflammatory cells at the tendon‐to‐bone interface. During the repair process, Sirius Red staining results at different time points indicated changes in collagen. Red and yellow indicated type I collagen, while green indicated type III collagen and statistical analysis was performed on the collagen types (Figure 9D,E). At week 2, both the BMP and BMRP groups exhibited slightly more type III collagen. As the repair progressed, the proportion of type I collagen in the BMP and BMRP groups gradually increased, and the orientation of the fiber arrangement became more organized. The collagen proportion at the injury site in the control group showed little change, with more type III collagen observed in the later stages of repair. Combining the results from HE staining (Figure 9F) and Masson staining, scores were assigned to the tendon‐to‐bone interfaces (Table S2, Supporting Information) and tendons (Table S3, Supporting Information) in different groups, with the BMRP group receiving the highest score, indicating the best repair effect (Figure 9G,H).

The early inflammatory response at the osteoporotic tendon‐to‐bone interface is closely related to the occurrence of scar healing. Increasing evidence suggests that a higher accumulation of M1 macrophages is detrimental to the repair.^[^ ^54^, ^55^, ^56^ ^]^ Tendon tissue is a dense connective tissue primarily composed of type I collagen, and an inflammatory response may lead to disorganized collagen regeneration, promoting the generation of type III collagen and resulting in decreased biomechanical properties.^[^ ^2^, ^32^, ^57^ ^]^ Both the BMP and BMRP groups may promote the generation of type III collagen in the early stages of repair. It has been reported that the synthesis rate of type III collagen in vivo is faster than that of type I collagen, and the formation of an extracellular matrix in the early stages of repair is beneficial for tissue recovery. Teriparatide and R‐PTH can effectively promote TDSCs to produce type I collagen. During the process of microstructural remodeling and functional reconstruction at the tendon‐to‐bone interface, type III collagen gradually transformed into the mechanically stronger type I collagen, thereby enhancing the strength and tensile capacity of the interface.

The BMRP scaffold has demonstrated significant potential for tendon‐to‐bone regeneration in osteoporotic conditions. However, further research is required to optimize its therapeutic efficacy and enhance its clinical applicability. Future investigations into tendon‐to‐bone interface scaffold materials should not only focus on advancing drug delivery systems, novel molecular designs, and multi‐scale structural optimization of biomimetic materials but also explore the underlying molecular mechanisms and cellular interactions in greater depth.

## 3 Conclusion

In summary, a tendon‐to‐bone biomimetic microstructured scaffold, utilizing decalcified mussel shells and fabricated via the ice‐templating method, was developed. When loaded with R‐PTH, the BMRP scaffold effectively repaired injured cells at the osteoporotic tendon‐to‐bone interface, promoted the migration of TDSCs, regulated bone homeostasis, and facilitated the remodeling of the interface microstructure, thereby promoting the regeneration and reconstruction of the osteoporotic tendon‐to‐bone interface. Furthermore, the effects of sustained stimulation with Teriparatide or R‐PTH on bone homeostasis at the osteoporotic tendon‐to‐bone interface were explored. Importantly, our newly developed R‐PTH, when applied locally, effectively avoids the excessive activation of osteoclasts caused by Teriparatide. This provides new perspectives and strategies for balancing bone homeostasis and regenerating the microstructure at the osteoporotic tendon‐to‐bone interface.

## 4 Experimental Section

### Design and Synthesis of R‐PTH

The design of R‐PTH was based on the amino acid sequence of Teriparatide (PTH1‐34). The following steps were taken to create R‐PTH. The first two amino acids (positions 1 and 2) of the PTH(1‐34) sequence were removed, resulting in PTH(3‐34). The sequence from positions 29 to 34 of PTH(3‐34) was duplicated and appended to the end of the sequence. The final amino acid sequence of R‐PTH is: SEIQLMHNLGKHLNSMERVEWLRKKLQDVHNYQDVHNY. Both PTH(1‐34) and R‐PTH were synthesized with the assistance of Mecklin Co., Ltd. (Shanghai, China).

### Experimental Animals

A total of 72 female SD rats (12 weeks old, weighing 220 ± 10 g) were used. All animals were purchased from the Experimental Animal Center of Southern Medical University and the study was approved by the Animal Ethics Committee of Southern Medical University (Approval No.: IACUC‐LAC‐20240514‐001).

### Materials

Sodium polyacrylate (PAA), sodium alginate (SA), acrylic acid (AA), 1‐Ethyl‐3‐(3‐dimethylaminopropyl) carbodiimide (EDC), N‐Hydroxysuccinimide (NHS), and ammonium persulfate (APS) were purchased from Mecklin Co., Ltd. (Shanghai, China). Gallic acid (GA) was purchased from SIGMA Co., Ltd. (USA). *N*, *N*″‐Methylenebis(acrylamide) (BIS) and *N*, *N*, *N*″,*N*′‐Tetramethylethylenediamine (TEMED) were purchased from Aladdin Co., Ltd. (Shanghai, China). UIO‐66‐NH2 was purchased from Yuanye Co., Ltd. (Shanghai, China).

### Synthesis of PAA‐NHS, OSA‐GA, and MOF‐Peptide

In a 100 mL solution of 2.5% PAA, NHS (2 g) was added and stirred in an ice bath. EDC•HCl (3 g) was added and stirred at room temperature overnight. Dialyzed for three days, then freeze‐dried to obtain PAA‐NHS. In a 100 mL solution of 2% SA, sodium periodate (1 g) was added and stirred in the dark at room temperature for 12 h. Ethylene glycol was added to terminate the reaction, dialyzed for three days, and freeze‐dried to obtain oxidized SA (OSA). In a 100 mL solution of 1% OSA, added 500 µL of 30% hydrogen peroxide, ascorbic acid (0.216 g), and nitrogen gas, and stirred for 30 min. GA (1 g) was added and stirred in the dark at room temperature for 24 h. Dialyzed and freeze‐dried to obtain OSA‐GA. MOF (2 mg) was added to 2 mL of peptide solution (500 µg mL^−1^) and ultrasonicated and stirred for 1 h. Centrifuged at 2000 rpm, discarded the supernatant, and then freeze‐dried to obtain the MOF‐peptide.

### Preparation of Biomimetic Tendon‐to‐Bone Microstructured Scaffolds

The cleaned mussel shell was washed and soaked it in a 10% nitric acid solution for 48 h. The shell was removed, washed it with deionized water, and freeze‐dried to obtain the polysaccharide scaffold. Dissolved OSA‐GA (10 mg), PAA‐NHS (5 mg), AA (168 mg), and BIS (1.2 mg) in 1 mL of PBS to obtain the pre‐gel. Added APS (2 mg) and TEMED (1 µL), mixed thoroughly, and injected the mixture into the polysaccharide scaffold. Placed the scaffold in a water bath at 60 °C for 1 min, then immediately transferred it to an ice template device and kept it for 48 h. Freeze‐dried the scaffold to obtain the BM scaffold. In 1 mL of the pre‐gel, added 0.2 mg of MOF‐Teriparatide. Followed the same subsequent steps to prepare the biomimetic microstructured scaffold loaded with Teriparatide (BMP). In 1 mL of the pre‐gel, added MOF‐RPTH (0mg, 0.01, 0.1, 0.2, 0.4 mg). Followed the same subsequent steps to prepare the biomimetic microstructured scaffold loaded with R‐PTH (BMRP).

### Cell Culture

BMSCs and bone marrow‐derived macrophages were isolated from the femurs of SD rats and cultured in F12‐Dulbecco's Modified Eagle's Medium (Gibco, USA) and minimum essential medium alpha (Gibco, USA), respectively. TDSCs were isolated from the Achilles tendons of SD rats and cultured in Dulbecco's Modified Eagle's Medium. BMSCs and TDSCs were identified through flow cytometry (Figure S18, Supporting Information). Mouse‐derived macrophage (RAW264.7) and mouse‐derived osteoblasts (MC3T3‐E1) were purchased from the Cell Bank of the Chinese Academy of Sciences Shanghai Institute of Life Sciences and cultured in Dulbecco's Modified Eagle's Medium for RAW264.7 and MC3T3‐E1‐specific medium (Procell, China) for MC3T3‐E1. All media contained 10% fetal bovine serum (FBS; Gibco, USA) and 1% penicillin‐streptomycin (P/S; Gibco, USA).

### TEM and SEM

Samples were prepared and observed using TEM (Bruker Nano GmbH Berlin, Germany) to examine the microstructure and elemental distribution of MOF and MOF‐peptide. The microstructure of the biomimetic microstructured scaffold was observed using SEM (ZEISS ULTRA 55, Japan).

### FTIR

Samples were prepared and measured using a Fourier transform infrared spectrometer (Thermo Scientific Nicolet iS50).

### Degradation Experiment

The biomimetic microstructured scaffold was immersed in PBS or collagenase solution (Type III collagenase: 0.5 mg mL^−1^). At different time points, the scaffolds were removed, freeze‐dried, and weighed to plot the degradation curve.

### Sustained Release Experiment

Peptide or MOF‐peptide was directly mixed into the pre‐gel to prepare the scaffold. The sustained release performance of the scaffolds with and without MOF was compared. The scaffolds were immersed in PBS, and samples were taken at different time points. Measured the absorbance peak at 280 nm using a micro‐UV spectrophotometer to calculate the release rate of the peptide and plot the release curve.

### Live/Dead Staining

At different time points, stained the cells on the scaffold using a Calcein‐AM/PI staining kit. Prepared the working solution (AM:PI: PBS = 2:1:1000), incubated the cells on the scaffold for 10 minutes, and observed under a fluorescence microscope (IX83 Olympus), and took photos.

### Antioxidant Stress Injury Experiment

MC3T3‐E1 cells were cultivated in a medium containing 100 µmol L^−1^ hydrogen peroxide for 12 h. Then, the injured MC3T3‐E1 cells were transferred to different gel scaffolds and cultured for 48 hours. Stained the cells using a DCFH‐DA kit, observed under a fluorescence microscope, and took photos. Digested the cells, centrifuged, collected the cells, and analyzed the fluorescence intensity using a high‐dimensional flow cytometer (BD LSRFortessa X‐20).

### Macrophage Polarization Experiment

RAW264.7 cells were treated with 100 ng mL^−1^ lipopolysaccharide (LPS) and 10 ng mL^−1^ interferon‐gamma (IFN‐γ) for 24 h to induce M1‐type macrophages. Then, the M1‐type macrophages were seeded on different scaffolds and cultured for 48 h. Cells were fixed and immunofluorescence staining was performed, observed under a fluorescence microscope, and photos were taken.

### Scratch Assay

TDSCs were seeded in the lower chamber of Transwell plates and different scaffolds were placed in the upper chamber. When the cells reached confluence, created scratches using a pipette tip and washed with PBS. Replaced with serum‐free medium and observed the scratch closure at different time points under a microscope, taking photos to record the results.

### Transwell Assay

TDSCs were seeded in the upper chamber of Transwell plates and different scaffolds were placed in the lower chamber. After culturing for 24 h, the cells were fixed with 4% paraformaldehyde. Removed the cells from the upper chamber, stained with crystal violet, observed the TDSCs that have migrated through the membrane, and took photos to record the results.

### ALP Staining

BMSCs were seeded on different scaffolds and cultured in an osteogenic induction medium. At different time points, the cells were fixed with 4% paraformaldehyde and stained using an ALP staining kit (Beyotime, China) at room temperature for 2 h. Observed under a microscope and took photos. Performed semi‐quantitative analysis of ALP staining using ImageJ software.

### ARS Staining

BMSCs were seeded on different scaffolds and cultured in an osteogenic induction medium. At different time points, fixed the cells with 4% paraformaldehyde and stained using ARS staining solution (Solarbio, China) at room temperature for 20 min. Observed under a microscope and took photos. The calcium nodules were dissolved in 10% cetylpyridinium chloride and the absorbance of the solution was measured at 540 nm using a microplate reader (Thermo Scientific, USA) for semi‐quantitative analysis of the calcium nodules.

### BMSCs and Macrophages Co‐Culture System

BMSCs were seeded on the scaffolds and placed them in the upper chamber of Transwell plates. In the lower chamber, rat bone marrow‐derived macrophages were seeded. Added 50 ng mL^−1^ RANKL and 25 ng mL^−1^ macrophage colony‐stimulating factor (M‐CSF) to the culture medium to induce osteoclast differentiation. On the third day, the cells were fixed with 4% paraformaldehyde and performed TRAP staining or immunofluorescence staining.

### RNA Extraction and Quantitative Real‐Time PCR

Co‐cultured the cells and scaffolds in Transwell plates. At different time points, extracted total RNA using the TRIzol method. cDNA was synthesized using the PrimeScript RT Reagent Kit (Takara, Japan). Next, qPCR reactions were performed using the StepOnePlus real‐time PCR system (Applied Biosystems, USA). The results were calculated using the 2^−ΔΔCT^ method. All primer sequences are listed in Table S4 (Supporting Information).

### Western Blot

Cells were lysed using RIPA buffer (Beyotime, China) and centrifuged at 4 °C to obtain protein samples. Protein concentration was determined using a BCA kit (Beyotime, China). Samples were lysed with RIPA buffer (Beyotime, China) and centrifuged at 1623 × *g* for 15 min at 4 °C. Protein concentration was measured using a BCA kit (Beyotime, China). Proteins were separated by SDS‐PAGE gel electrophoresis and then transferred to PVDF membranes. The membranes were blocked with 5% skim milk and incubated with primary antibodies, followed by secondary antibodies. Visualization was performed using a chemiluminescent imaging system (Bio‐Rad). Finally, protein expression levels were analyzed and quantified using ImageJ software.

### Immunofluorescence

Cells were fixed with 4% paraformaldehyde, washed, and permeabilized with 0.2% Triton X‐100. The cells were then blocked with 2% BSA and incubated with primary antibodies overnight at 4 °C. Fluorescent secondary antibodies were added and incubated in the dark. DAPI was added as a nuclear counterstain. The samples were observed and photographed under a laser confocal microscope (Zeiss LSM900). Primary antibodies used were: anti‐CD86 (ab239075, abcam), anti‐CD206 (ab64693, abcam), anti‐COL I (bsm‐33401 M, bioss), and anti‐TNMD (bs‐7525R, bioss).

### RNA Sequencing

BMSCs were seeded on the scaffolds and cultured for three days. Total RNA was extracted using the TRIzol method and mRNA was purified. Sequencing libraries were generated and sequenced on the Illumina NovaSeq 6000 platform to produce raw data. The raw data were aligned to the Rattus norvegicus genome using HISAT2. DEGs were screened with criteria of log2|fold change| > 1 and adjusted *p*‐value < 0.05. Functional enrichment analysis of significantly DEGs was performed using clusterProfiler for GO and KEGG pathway categories, with functions having a *p*‐value < 0.05 considered significant. GSEA was also performed using clusterProfiler.

### Construction of Osteoporotic RCT Model

Before the experiment, female SD rats were preliminarily screened to ensure consistent baseline physiological status. Postmenopausal osteoporosis was induced using ovariectomy (OVX): under isoflurane anesthesia, a midline incision was made on the back, and the ovaries and fallopian tubes were exposed through blunt dissection, ligated with 4‐0 silk thread, and excised. In the sham‐operated control group, only laparotomy was performed without ovary removal to exclude the effects of surgery itself. The muscle and skin layers were then sutured in layers, and the rats were maintained under standardized conditions (controlled temperature and humidity, fixed light cycles, and standard diet) for 12 weeks. Micro‐CT measurements confirmed a significant decrease in bone density, validating the successful induction of osteoporosis. The experimental design strictly included a sham‐operated control group to differentiate the effects of surgical stress from those of estrogen deficiency on bone metabolism. For the osteoporotic rats, isoflurane inhalation anesthesia was administered, and the shoulder area was shaved. The surgical area was disinfected with iodine, and an incision was made anterior to the shoulder. Soft tissues were bluntly dissected to expose the supraspinatus muscle. The supraspinatus tendon was sectioned at its enthesis on the humerus, and the tendon ends were sutured. The scaffold was implanted at the enthesis, and the tendon ends were sutured back to the enthesis. The muscle and skin layers were sutured sequentially.

### Gait Analysis

After RCR in osteoporotic SD rats, at different time points, blue dye was applied to the healthy side paw, and red dye was applied to the surgical side paw. The rats were placed on a runway and guided to walk naturally to the other end. Footprints were recorded, and various gait parameters were measured. The asymmetry index (AI) was calculated as follows: AI (%) = [(NS − ES) / (NS + ES)]/0.5 × 100, where NS is the measurement value of the normal side and ES is the measurement value of the surgical side.

### MRI Examination

At different time points post‐surgery, MRI examinations were performed. The rats were anesthetized with isoflurane inhalation, and images of the shoulder joint were acquired using a small animal magnetic resonance imaging system (PharmaScan 70/16 US, Bruker) with a field strength of 7.0T.

### Biomechanical Testing

The distal end of the humerus and the proximal end of the supraspinatus were fixed on a universal testing machine (Instron 6800, US), and the interface was slowly stretched to test the maximum load it could withstand.

### Micro‐CT

The excised humerus was immersed in 4% paraformaldehyde and fixed for 24 h. The samples were then scanned using a micro‐CT system (Bruker, Belgium). The data were reconstructed in three dimensions using AVIZO software for analysis.

### TRAP Staining, Masson Staining, HE Staining, and Sirius Red Staining

Excised samples were fixed in 4% paraformaldehyde for 24 h and decalcified using EDTA decalcification solution. The decalcified samples were embedded in paraffin and sectioned into 4 µm thick slices. The sections were deparaffinized and rehydrated. For TRAP staining, a working solution was prepared using a TRAP staining kit (Sigma, USA), and the sections were stained. Nuclei were counterstained with methyl green and the slides were mounted with neutral resin. For Masson and HE staining, the sections were stained using Masson staining kits (Solarbio, China) and HE staining kits (Solarbio, China), respectively. After staining, the slides were mounted with neutral resin and observed under a microscope, with photographs taken. For Sirius Red staining, the sections were stained with Sirius Red dye, dehydrated, and mounted. The slides were observed under a polarized light microscope and photographed.

### Statistical Analysis

All statistical analyses were performed using SPSS (version 26.0, USA). Data were expressed as mean ± standard deviation (SD). One‐way ANOVA followed by Tukey's multiple comparisons test was used. *P‐*values less than 0.05 were considered statistically significant.

## Conflict of Interest

The authors declare no conflict of interest.

## Supporting information



Supporting Information

## Data Availability

The data that support the findings of this study are available from the corresponding author upon reasonable request.
