# Supplementary material for: Biomimetic Microstructured Scaffold with Release of Re‐Modified Teriparatide for Osteoporotic Tendon‐to‐Bone Regeneration via Balancing Bone Homeostasis
Source: Adv Sci (Weinh). 2025 Mar 17;12(18):2500144. doi: 10.1002/advs.202500144 (PMC12079530; doi:10.1002/advs.202500144)
Supplement: Supplementary file 1 — Supporting Information [file ADVS-12-2500144-s001.docx]

**Supporting Information**

**Biomimetic Microstructured Scaffold with Release of Re-modified Teriparatide for Osteoporotic** **Tendon-to-Bone Regeneration via Balancing Bone Homeostasis**

Chengzhong Xu^a,b, ‡^, Sijie Qiu^c, ‡^, Zhigen Yuan^a, ‡^, Chongyin Qiu^b^, Wenyu Xu^a^, Jialiang Guo^a^, Gen Wen^a^, Shuai Liu^b^, Wenjuan Yan^c^, Haibing Xu^d,^*, Honghao Hou^b,^*, Dehong Yang^a,^*

^a^Department of Orthopaedics-Spine Surgery, Nanfang Hospital, Southern Medical University,1838 North Guangzhou Ave, Guangzhou 510515, P.R. China.

^b^Guangdong Provincial Key Laboratory of Construction and Detection in Tissue Engineering, School of Basic Medical Sciences, Southern Medical University, Guangzhou 510515, P. R. China.

^c^Department of Stomatology, Nanfang Hospital, Southern Medical University, Guangzhou, 510515, P.R. China.

^d^ Guangdong-Hong Kong-Macao Greater Bay Area Center for Brain Science and Brain-Inspired Intelligence, Department of Neurobiology, School of Basic Medical Sciences, Southern Medical University, Guangzhou 510515, P.R. China.

E-mail: haibingxu@smu.edu.cn (H. Xu); hhou2019@smu.edu.cn (H. Hou); yangdehong@smu.edu.cn (D. Yang)

*^‡^*These authors contributed equally to the work.


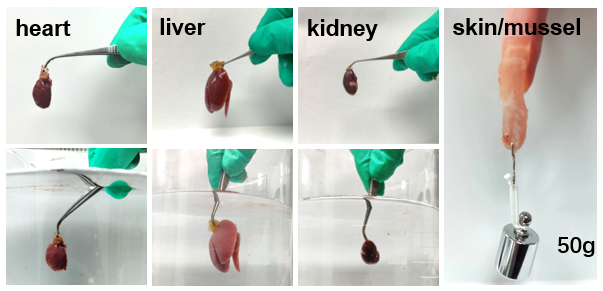


Figure S1. Images of the adhesive performance for biomimetic microstructured hydrogel scaffolds on different tissues.


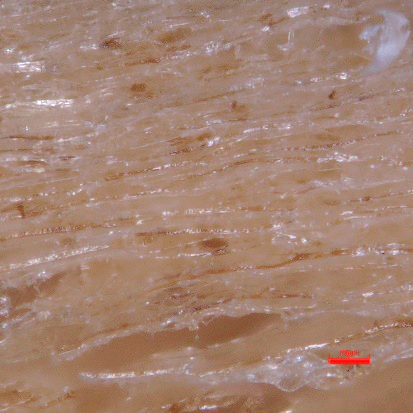

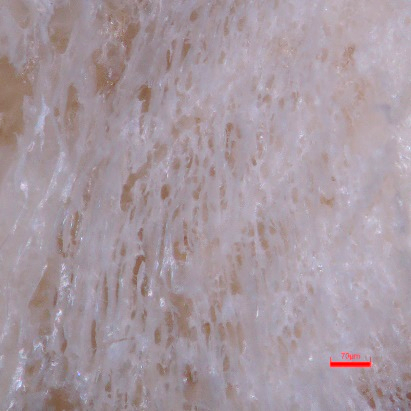


Figure S2. Ultra-depth microscope images of biomimetic microstructured hydrogel scaffolds. Scale bar: 70μm.


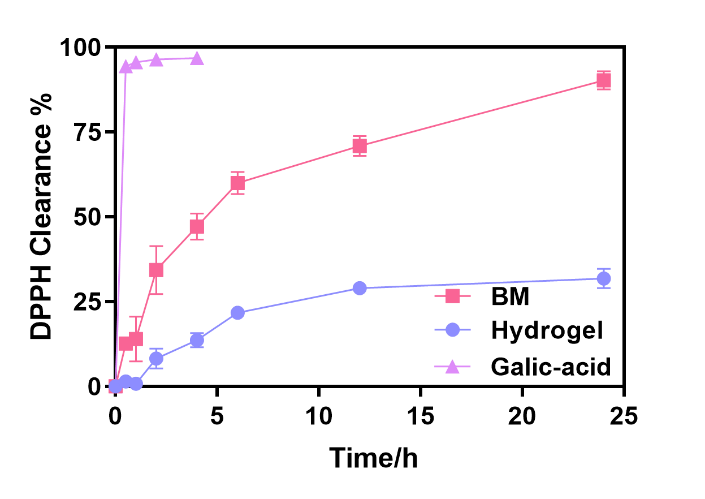


Figure S3. DPPH scavenging performance by biomimetic microstructured scaffolds. n=3.


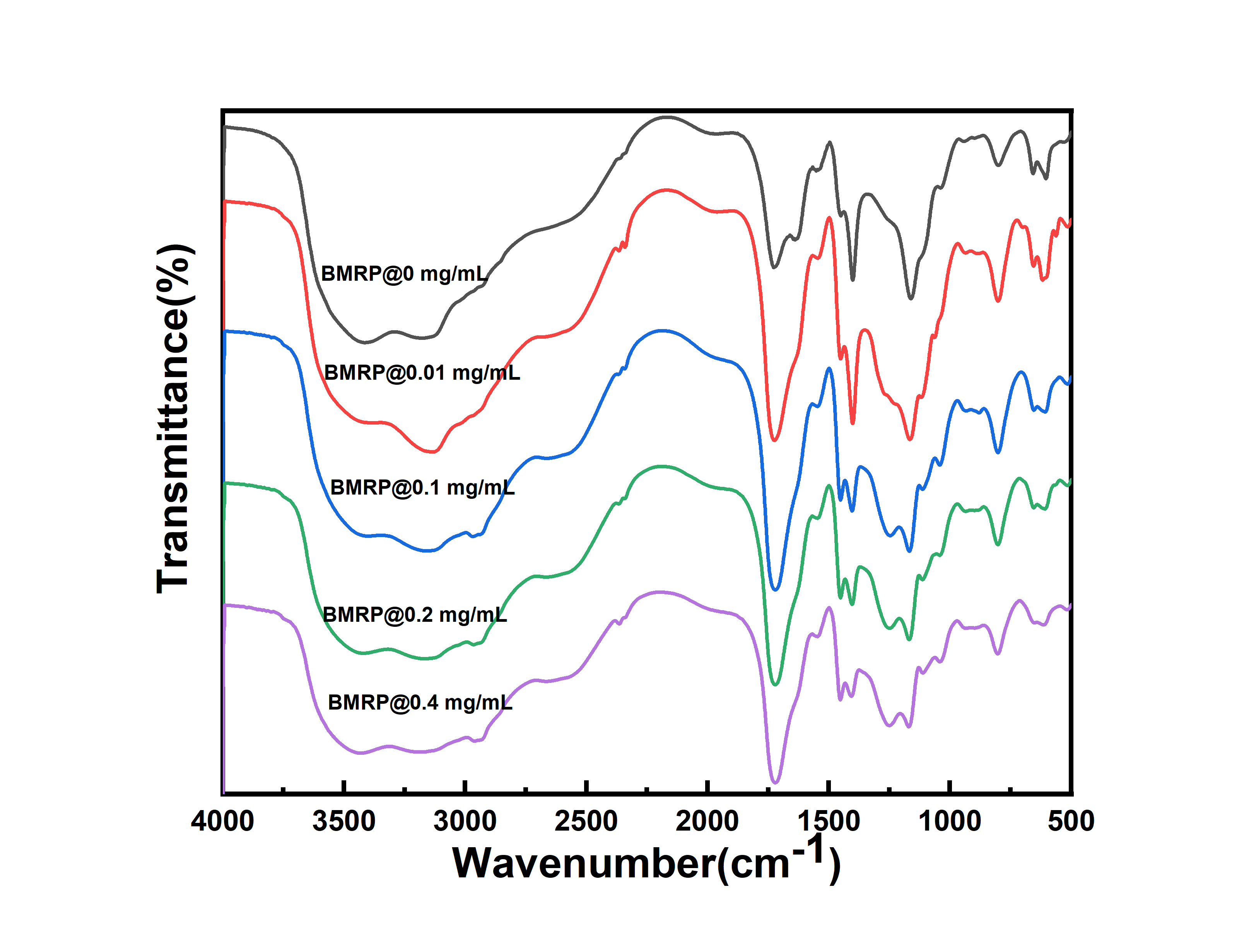


Figure S4. FTIR spectra of scaffolds loaded with different concentrations of MOF-RPTH.


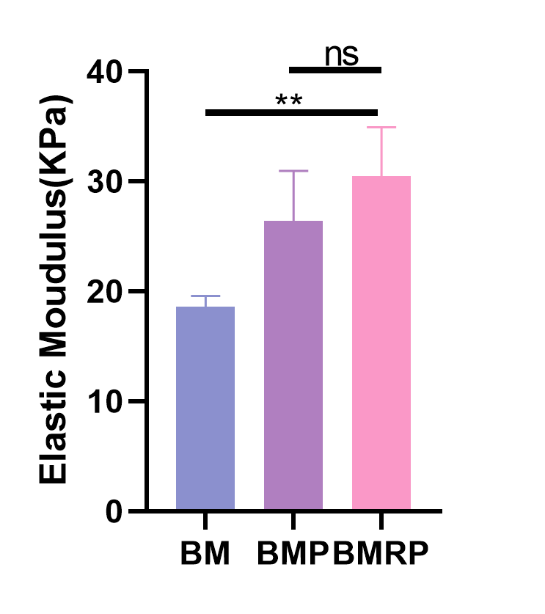


Figure S5. Compression modulus of different scaffolds. n=3. *ns P > 0.05, **P < 0.01*


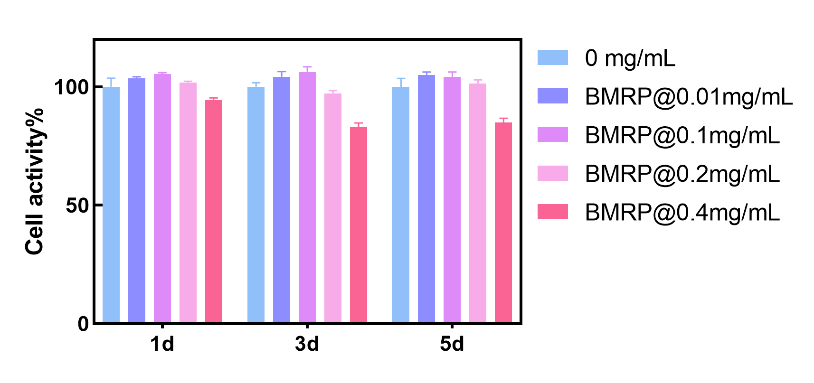


Figure S6. Viability of MC3T3-E1 cells on biomimetic microstructured scaffolds loaded with different concentrations of MOF-RPTH at days 1, 3, and 5. n=3.


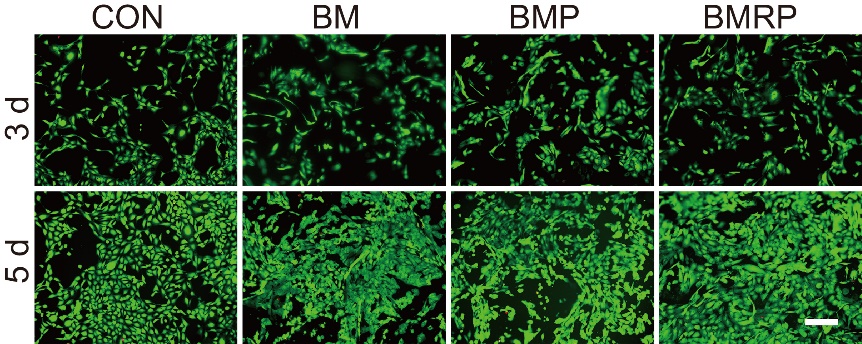


Figure S7. Live/Dead staining of MC3T3-E1 on different scaffolds at days 3 and 5. Scale bar: 100μm


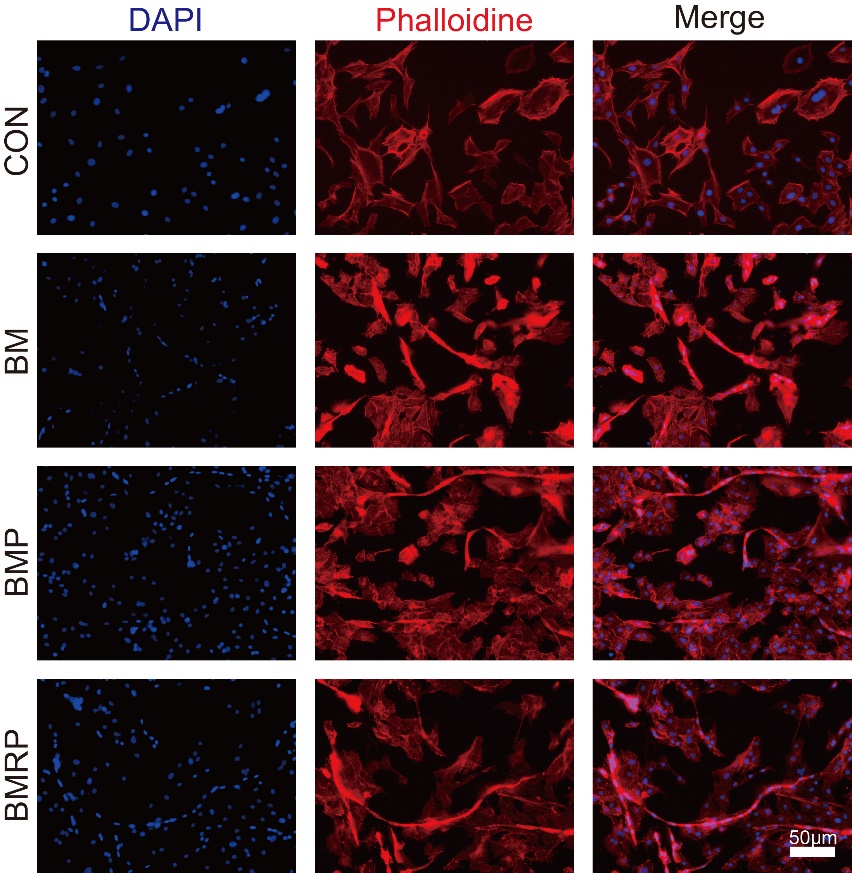


Figure S8. Phalloidin (red) immunofluorescence staining of MC3T3-E1 on different scaffolds at day 3. Scale bar: 50μm


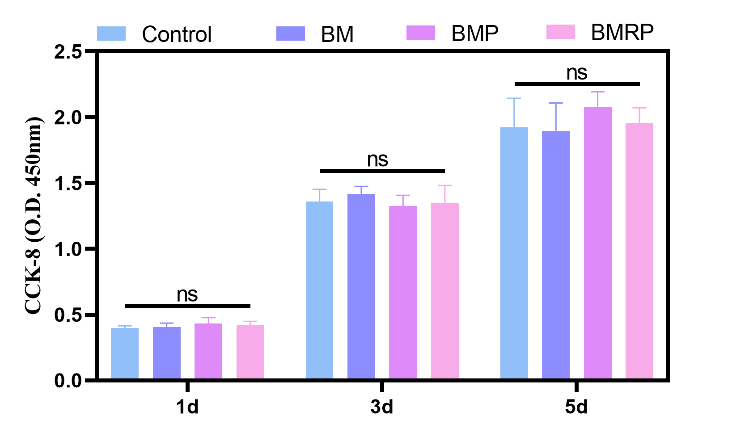


Figure S9. CCK-8 assay of MC3T3-E1 on different scaffolds at days 1, 3, and 5. n=3. *P > 0.05*


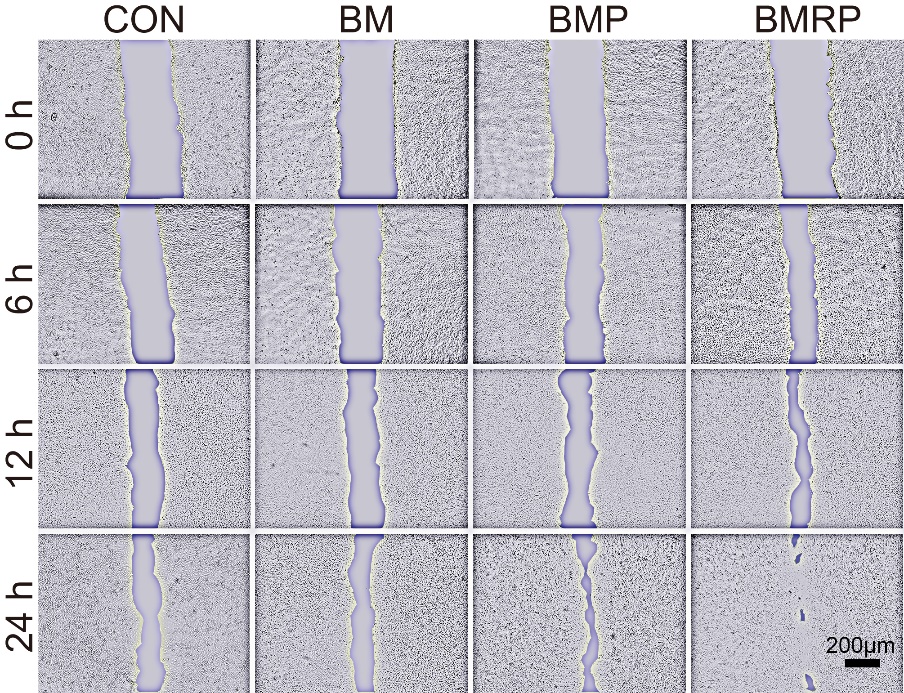


Figure S10. Effect of different scaffolds on the migration of TDSCs. Scale bar: 200μm


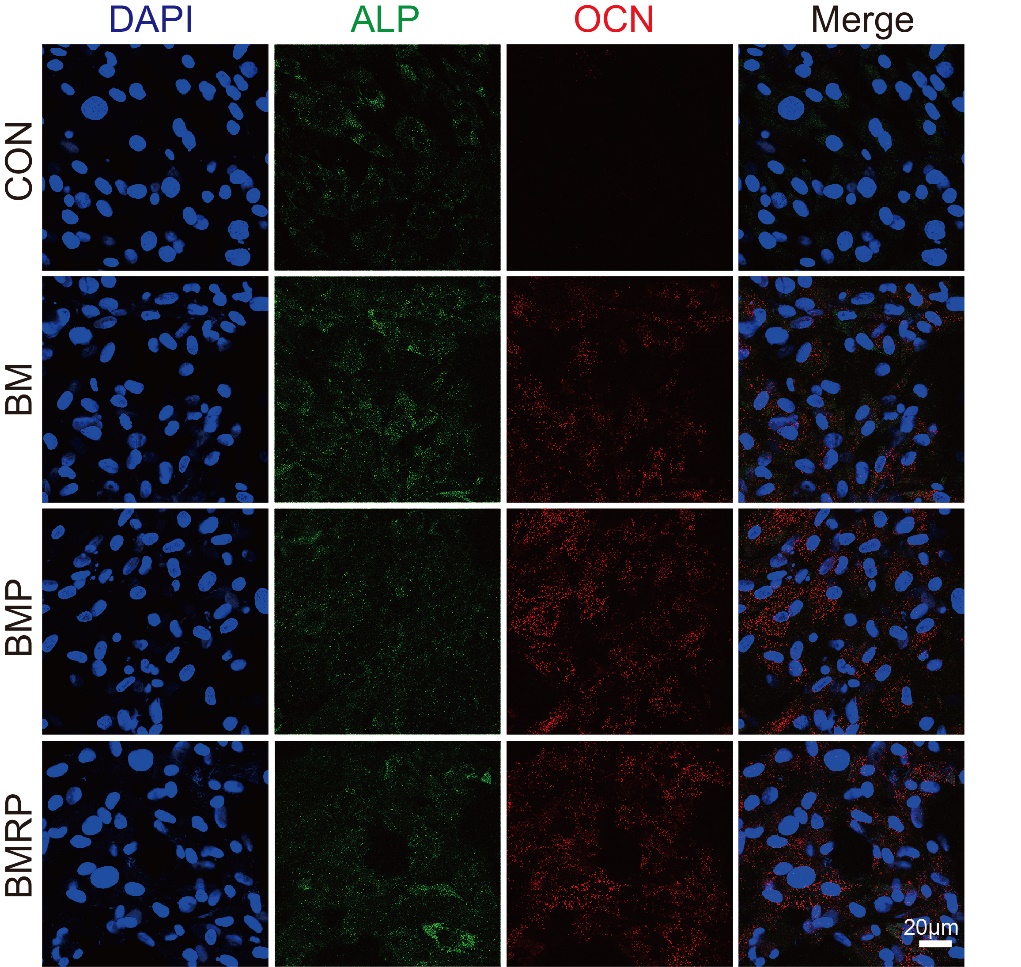


Figure S11. Immunofluorescence staining of ALP (green), OCN (red), and DAPI (blue) in BMSCs on different scaffolds at day 1. Scale bar: 20μm


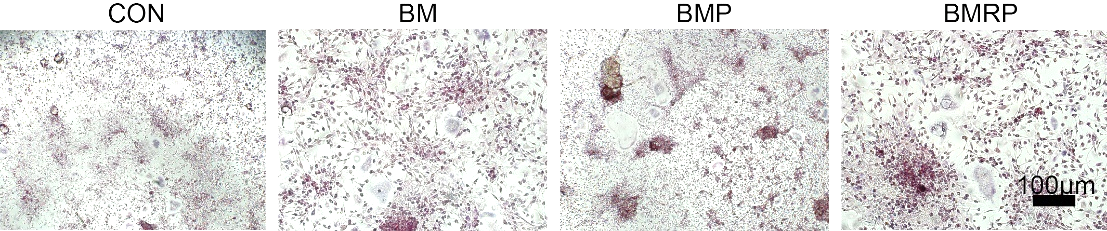


Figure S12. The differentiation of bone marrow-derived macrophages into osteoclasts on different scaffolds, with TRAP staining on day 3. Scale bar: 100μm


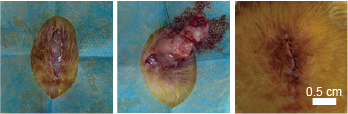


Figure S13. Bilateral ovariectomy surgery in female SD rats. Scale bar: 0.5 cm


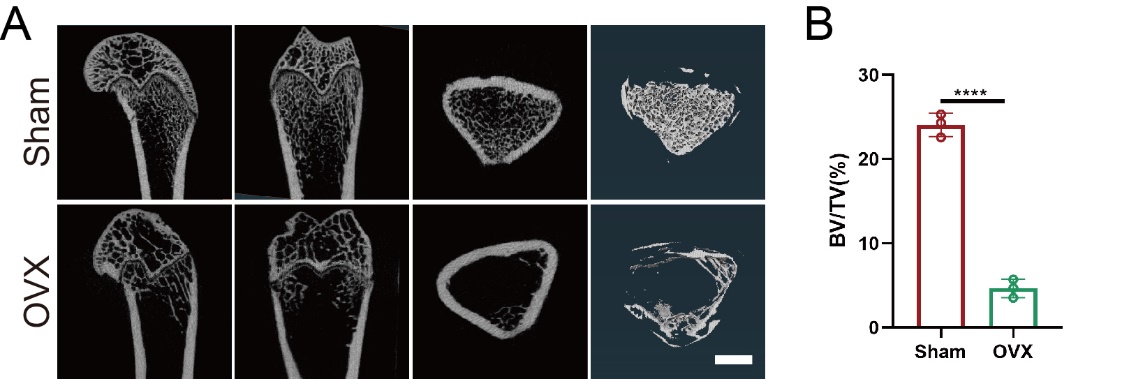


Figure S14. The successful establishment of an osteoporosis model in SD rats was confirmed by Micro-CT images (A) and statistical analysis of BV/TV (B). Scale bar: 2 mm. *****P < 0.0001*


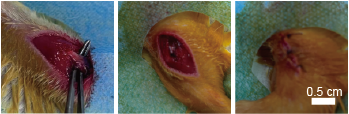


Figure S15. Establishment of an osteoporotic RCT SD rat model. Scale bar: 0.5 cm


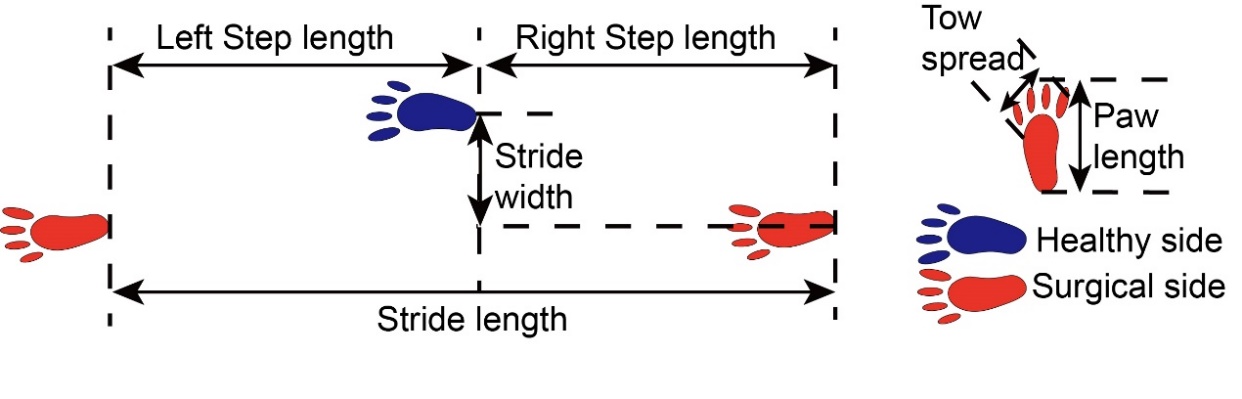


Figure S16. Schematic diagram of gait analysis measurement parameters.


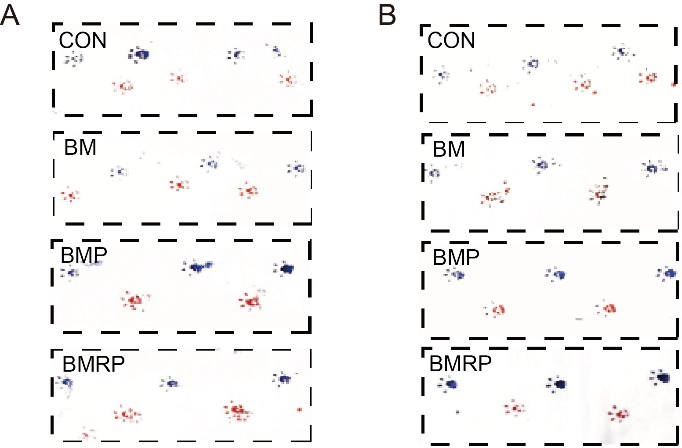


Figure S17. Footprint from gait analysis at 2 (A) and 4 (B) weeks post-surgery in the osteoporotic RCT Model.


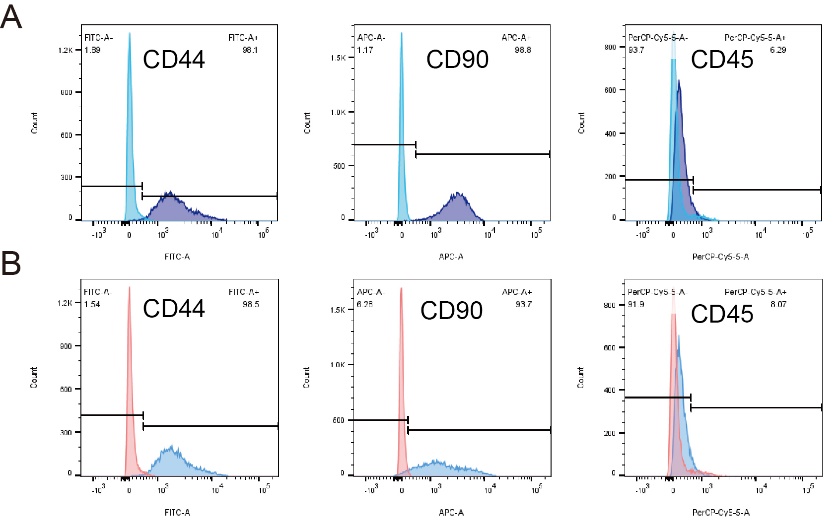


Figure S18. Flow cytometry identification of BMSCs (A) and TDSCs (B).

**Table S1. Tendon-to-bone interface evaluation (MRI grade)**

| **Grade** | | **Description** | |
| --- | --- | --- | --- |
| I  II  III  IV  V | Signal similar to tendon  Signal great than tendon but less than muscle  Signal similar to muscle  Signal greater than muscle but less than joint fluid  Signal similar to joint fluid | |  |

**Table S2. The histologic scoring of tendon-to-bone healing**

| **Items** | **Score** | | |
| --- | --- | --- | --- |
| 1. Cellular morphologic characteristics of interface tissue  Fibrocartilage with mature cartilage cells comprising ≥ 50%  Fibrocartilage with mature cartilage cells comprising < 50%  Fibrous tissue with Sharpey-like fibers comprising ≥ 50%  Fibrous tissue with Sharpey-like fibers comprising < 50%  Only fibrovascular tissue  2. Extent of surrounding fibrocartilage tissue  Mostly surrounded (≥ 75%)  Moderately surrounded (50% to < 75%)  Partially surrounded (25% to < 50%)  Slightly surrounded (< 25%)  Not visible  3. Interface tissue transition from bone to tendon  Mostly indistinct (≥ 75%)  Moderately indistinct (50% to < 75%)  Partially indistinct (25% to < 50%)  Continuous but slightly indistinct (< 25%)  Discontinuous | | 4  3  2  1  0    4  3  2  1  0    4  3  2  1  0 |  |

Maximum total score 12

**Table S3. The Modified Tendon Histological Evaluation score for the regenerated tendon substance**

| **Items** | **Score** | | | |
| --- | --- | --- | --- | --- |
|  | 0 | 1 | 2 | 3 |
| Cell density | Severely increased | Moderately increased | Slightly increased | Normal pattern |
| Rounding of nuclei | Severely rounded | Moderately rounded | Slightly rounded | Long spindle shape |
| Cell arrangement | Severely disordered | Moderately disordered | Slightly disordered | Parallel |
| Fiber density | Severely loose | Moderately loose | Slightly loose | Compacted |
| Fiber structure | Severely fragmented | Moderately fragmented | Slightly fragmented | Continuous, long fiber |
| Fiber arrangement | Severely rounded | Moderately rounded | Slightly rounded | Parallel |
| Inflammation (area infiltrated by inflammatory cells), % | > 30 | 20-30 | 10-20 | > 10 |
| Increased vascularity, % | > 30 | 20-30 | 10-20 | > 10 |
| Maximum total score | 24 | | | |

**Table S4. The primer sequences used for RT-qPCR assays.**

| **Gene** |  | **Primer sequence** |
| --- | --- | --- |
| GAPDH | Forward primer | CAGCCGCATCTTCTTGTGC |
| GAPDH | Reverse primer | ATCCGTTCACACCGACCTTC |
| ALP | Forward primer | ACCTGACTGACCCTTCCCTCTC |
| ALP | Reverse primer | CAATCCTGCCTCCTTCCACTAGC |
| OCN | Forward primer | CCGTTTAGGGCATGTGTTGC |
| OCN | Reverse primer | CCGTCCATACTTTCGAGGCA |
| Osx | Forward primer | GCCTACTTACCCGTCTGACTTTGC |
| Osx | Reverse primer | CCCTCCAGTTGCCCACTATTGC |
| RANKL | Forward primer | GAGCGTACCTGCGGACTATC |
| RANKL | Reverse primer | AACAGGGAAGGGTTGGACAC |
| Trap | Forward primer | ATGACGCCAATGACAAGAGGTTCC |
| Trap | Reverse primer | TTGTGCCGAGACATTGCCAAGG |
| Ctsk | Forward primer | GCTATGTTCTCTTGGCTCGGAATAAG |
| Ctsk | Reverse primer | TGGCTGGCTTGAATCACATCTTG |
| MMP9 | Forward primer | CAAACCCTGCGTATTTCCATTCATC |
| MMP9 | Reverse primer | GATAACCATCCGAGCGACCTTTAG |
